# Supplementary figures and images for: Targeting human langerin promotes HIV-1 specific humoral immune responses
Source: PLoS Pathog. 2021 Jul 29;17(7):e1009749. doi: 10.1371/journal.ppat.1009749 (PMC8354475; doi:10.1371/journal.ppat.1009749)

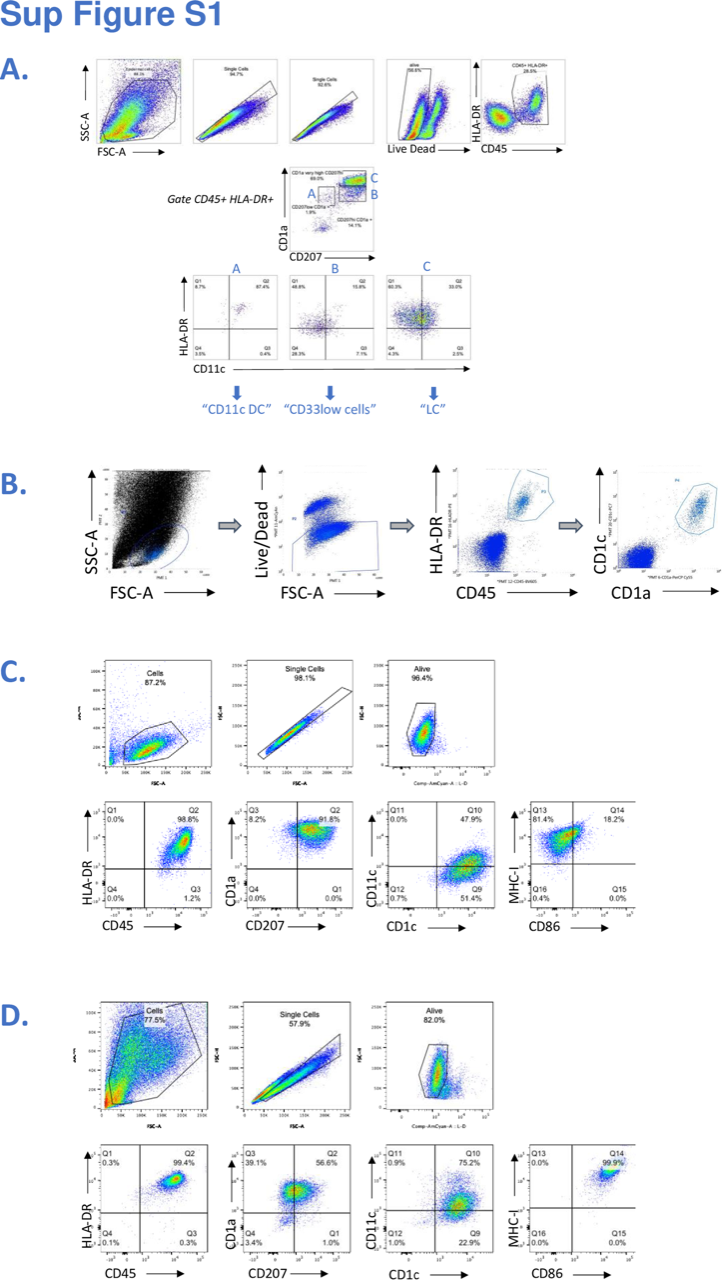

Supplement: S1 Fig — (A) Cell suspension from trypsin-digested human epidermis was stained and analyzed by flow cytometry. CD45+ HLA-DR+ populations showed 3 subpopulations, depending on the expression of CD207 and CD1a, namely populations A, B and C. Population “A”was CD11chi, “B” CD11clow and HLA-DRlow, whereas “C” was CD11clow and HLA-DR+. These skin epidermal populations was phenotypically close to epidermal myeloid populations already described [28]. (B) Skin epidermal cell suspension was sorting by the Influx cell sorter, using Live dead, CD45, HLA-DR, CD1a and CD1c markers. (C) Isolated steady-state skin cells were controlled by FACS. They show typical markers of LC, meaning CD207+, CD1ahi, CD1c+, CD11clow, and were not matured (MHC class I and CD86 low expression). (D) As comparison, cells migrated in culture from skin explants were phenotypically activated (MHC class Ihi, CD86hi) with expression of LC markers but at a lower level (CD1aint CD207int). (TIF) [file ppat.1009749.s001.tif]

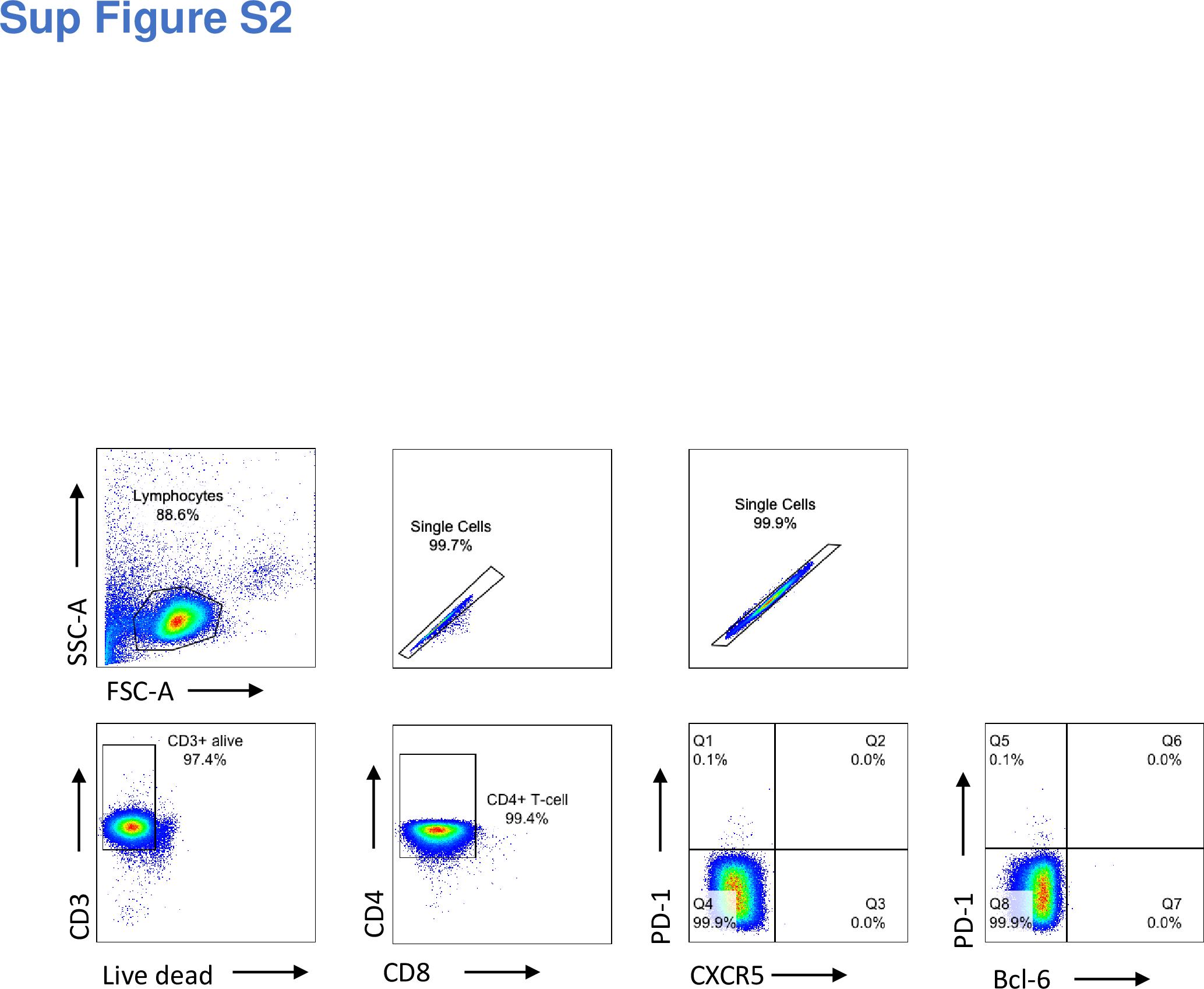

Supplement: S2 Fig — CD4+ naïve T cells from cord blood were controlled by FACS for the absence of Tfh cells, based on the expression of PD-1, CXCR5 and Bcl-6. (TIF) [file ppat.1009749.s002.tif]

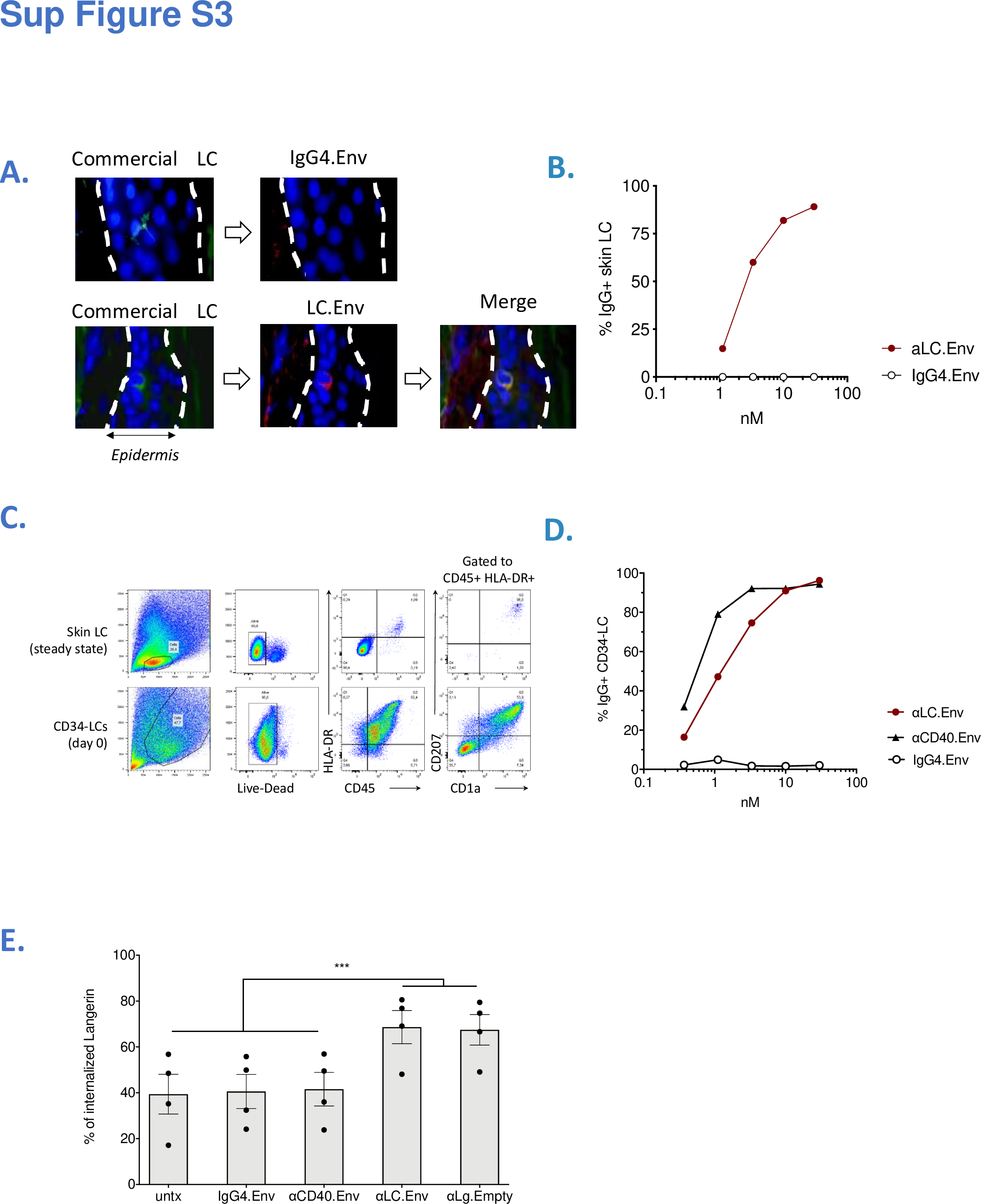

Supplement: S3 Fig — (A) Binding assay of αLC.Env mAb on epidermal LCs by immunofluorescence assay. Sections of frozen skin explants were treated with αLC.Env or IgG4.Env mAbs. Binding to skin LCs was controlled using a commercial anti-CD207 mAb (12D6). Pictures are representative of 4 different donors (magnification x20). Skin LCs are located within the epidermis and capture αLC.Env mAbs but not IgG4.Env. (B) Total epidermal cells were stained with phenotypical markers and incubated with serial dilution of αLC.Env (full red circle) or IgG4.Env (open circle). A specific binding of CD207+ cells was observed for αLC.Env in a dose-dependent manner by FACS. (C) Representative phenotypic analysis of steady state skin LCs and unmatured CD34-LCs at the end of the differentiation process and before CD1a sorting. Cells were characterized by high expression of CD1a and CD207. (D) As in A, binding assay of aLC.Env versus IgG4.Env on CD34-LC. Binding was comparable in freshly isolated skin LC and CD34-LC (IC50 = 3 nM and 1.4 nM, respectively). The binding of the αCD40.Env was tested in parallel (black triangles). (E) Internalization of the Langerin receptor. CD34-LC were incubated overnight (37°C) with αLC.Env, αLC.Empty, control mAbs (αCD40.Env, IgG4.Env) or cyclo-dextrin buffer (untx, untreated). We observed a significant internalization of Langerin 16h after treatment with αLC mAbs compared to control mAbs (P < 0.001), suggesting a rapid endocytosis of the antigen targeted to the Langerin receptor. Data are means (± SEM) of 4 donors. Two-way ANOVA with Dunnett’s correction were used for statistical analysis (***, P < 0.001). (TIF) [file ppat.1009749.s003.tif]

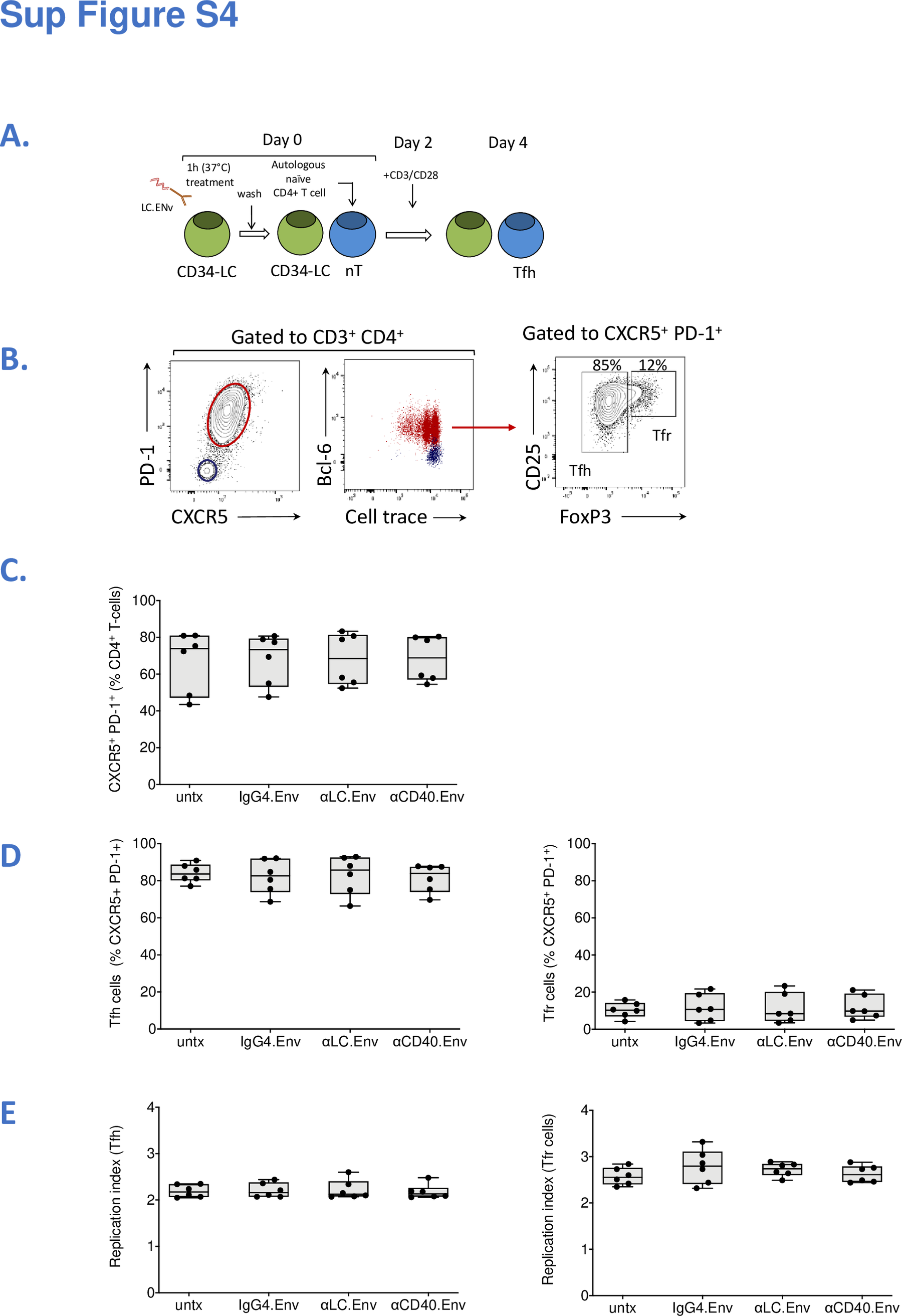

Supplement: S4 Fig — (A) Schematics of the procedure of the CD34-LC treated with αLC.Env or control mAbs and cultured with autologous naïve CD4+ T cells. (B) (left) Representative CD4+ T cell dot plots of one out of 6 donors. CXCR5+ PD-1+ population (red) was Bcl6+ and was proliferating as indicated by the loss of cell trace marker. (right) CXCR5+PD-1+ were either CD25+ FoxP3hi (Tfr cells) or FoxP3- (Tfh cells). (C) Percentages of CXCR5+PD-1+ among CD4+ T cells. (D) Percentages of Tfh vs Tfr cells among CXCR5+ PD-1+ cells after treating CD34-LC. (E) Replication indexes of Tfh cells and Tfr cells were calculated from the loss of cell trace marker. (TIF) [file ppat.1009749.s004.tif]

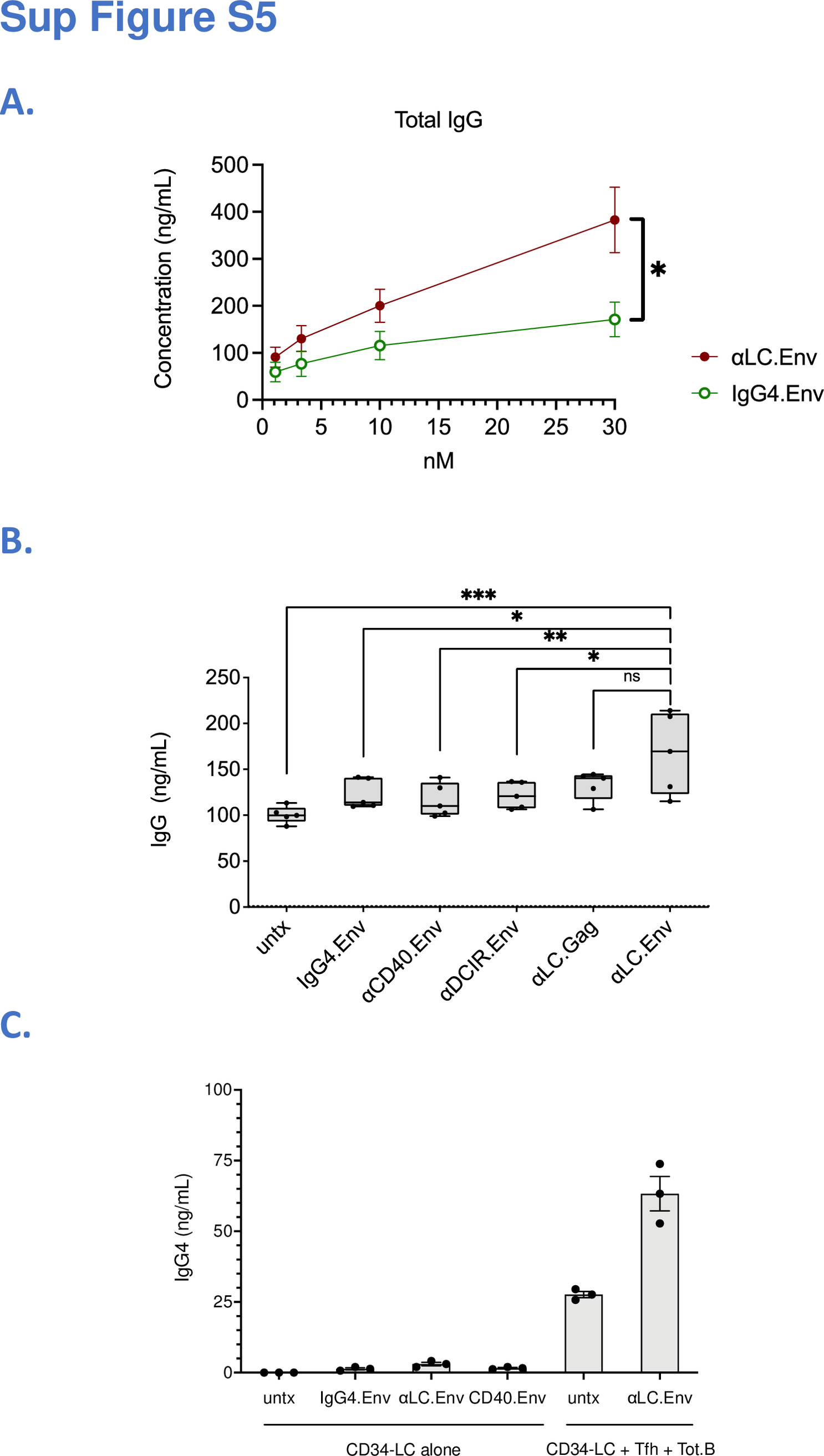

Supplement: S5 Fig — (A) Vaccine dose-dependent B cells responses of αLC.Env treated CD34-LC (plain circles) and IgG4.Env (open circles). (B) IgG production was compared to αLangerin mAbs fused with HIV-1 Gag antigen, or αDCIR, αCD40 and non-targeting IgG4 vehicles associated with the HIV-1 Env gp140z antigen. (C) IgG4 detected in cell co-cultures was not derived from the treatment of CD34-LC with IgG4-based vaccine. CD34-LC were treated with αLC.Env or control Abs and cultured 10 days without adding T- or B-cells (n = 3). As controls, CD34-LC were treated and cultured as in Fig 2 (n = 3). Concentrations of IgG4 in culture supernatant was measured by ELISA, using an anti-human IgG4 mAb (ThermoFisher) for coating. Non-parametric Kruskal-Wallis tests were performed (*; P < 0.05; **, P < 0.01; ***, P < 0.001; ns; non-significant). (TIF) [file ppat.1009749.s005.tif]

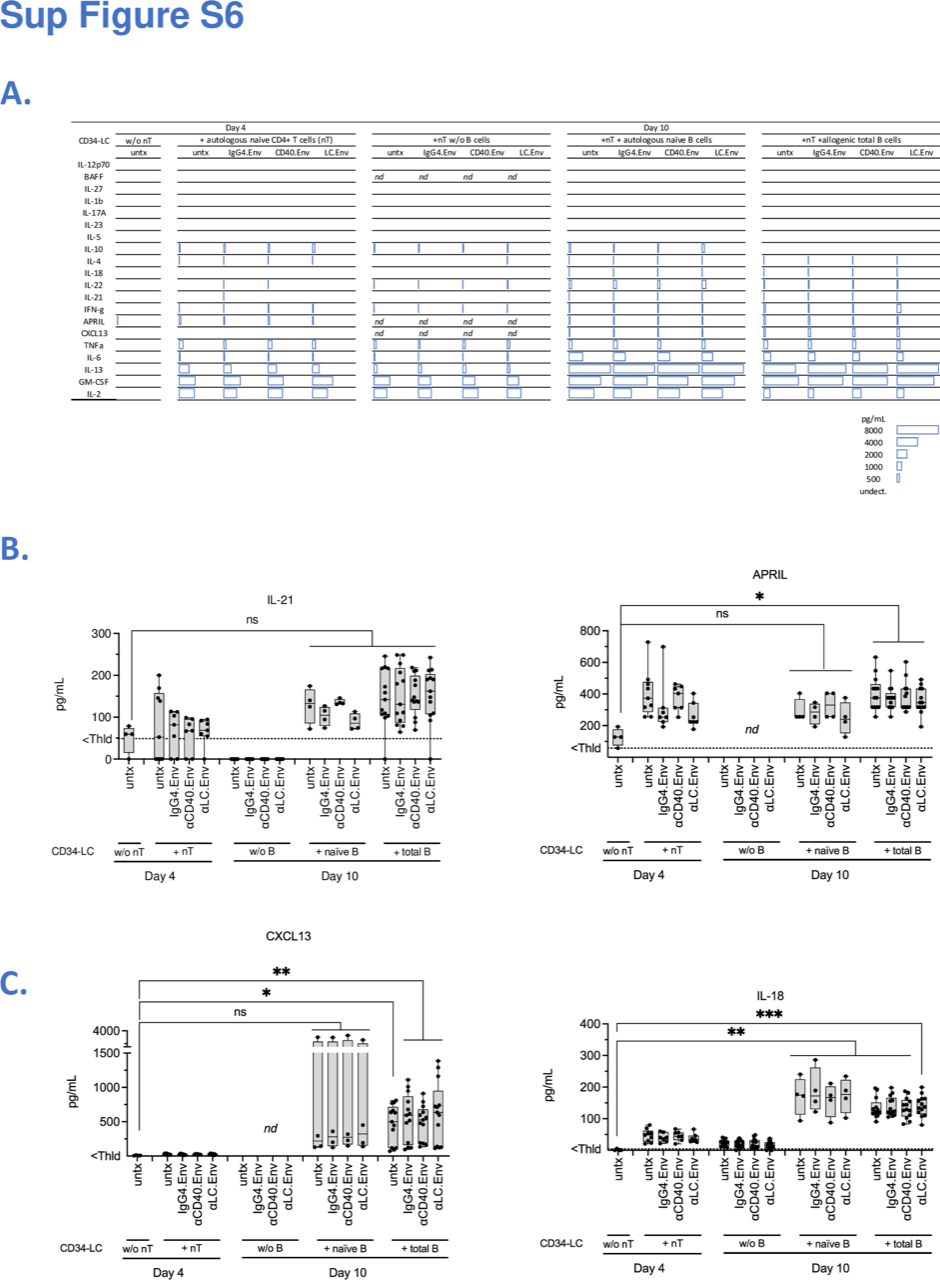

Supplement: S6 Fig — (A) As in Fig 3, multiplex beads analysis and comparison of culture supernatants at day 4 or 10 (as depicted in Fig 2A) with CD34-LC treated by αLC.Env, αCD40.Env or IgG4.Env, cultured alone (column 1), or with autologous naïve CD4+ T cells (column 2) for 4 days, and with or without (column 3) autologous naïve B cells (column 4) or total allogenic B cells (column 5). Color scale indicates median values of each condition (n = 4 to 9) and is expressed in pg/mL. Values of IL-21 and APRIL are detailed in (B), and IL-18 and CXCL13 in (C), respectively. Dotted line indicates threshold of detection. (TIF) [file ppat.1009749.s006.tif]

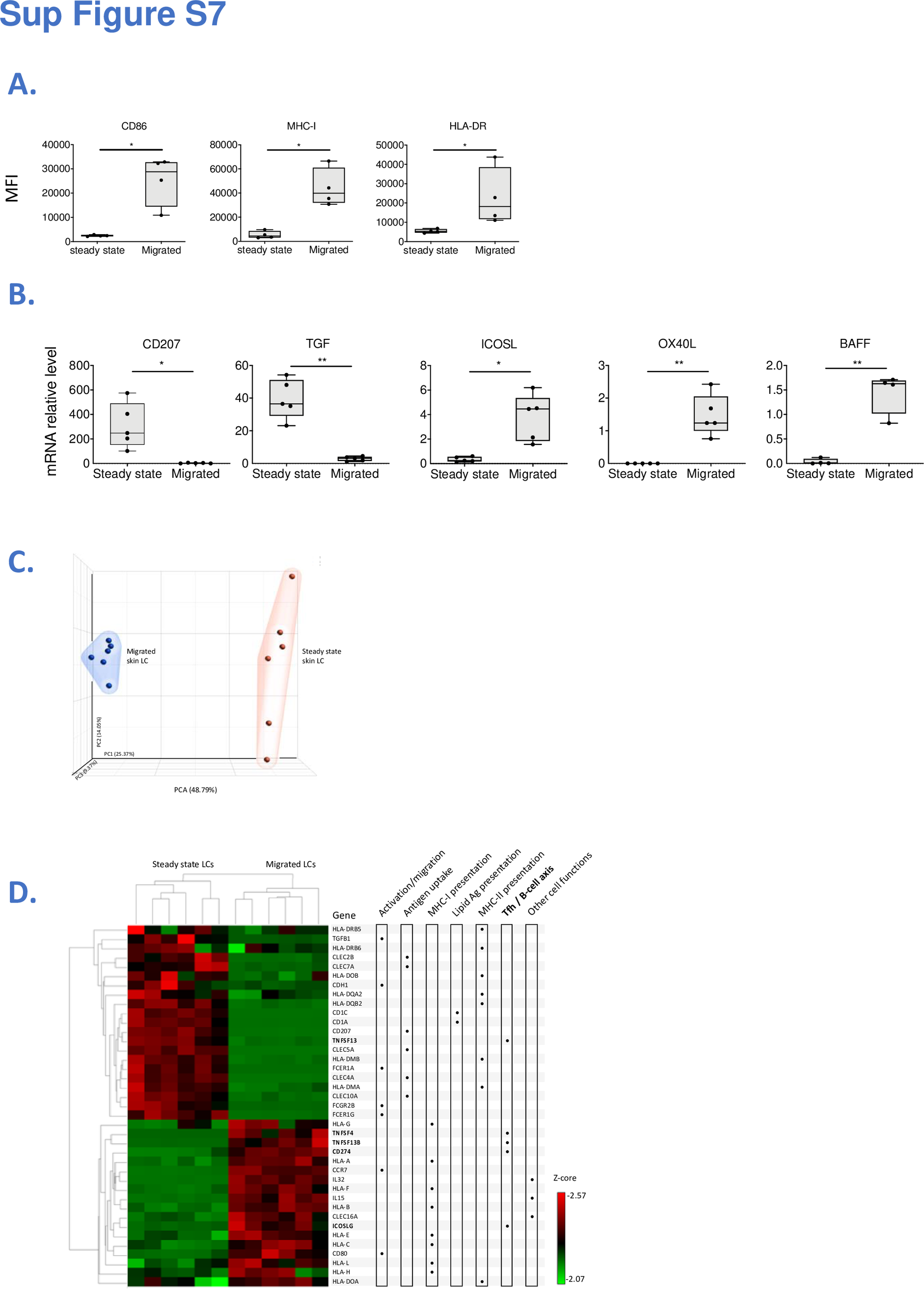

Supplement: S7 Fig — (A) Expression of maturation surface markers of skin LC was monitored by flow cytometry before and after migrating out of the epidermis (n = 4) (B) Real-time qPCR of skin LC transcripts. Total mRNAs from steady state epidermal cells vs migrated skin LC (n = 5) were isolated. Relative level expressions of transcripts associated with LC activation (CD207, TGFβ), T cell co-stimulation (ICOSL, OX40L), and gene genes potentially linked to the humoral immune response induction (BAFF) were quantified by qPCR. Statistics were obtained using the non-parametric Mann-Whitney test (*, P < 0.05; **, P < 0.01; ns, non-significant). (C) RNAseq analysis on primary skin LC. Epidermal LC of 6 healthy donors were isolated either at steady state or after 3 days of migration in culture. Unsupervised PCA of the transcriptional analysis revealed two distinct populations. (D) Modulation of the expression of genes associated with Tfh/B cell signaling pathway, antigen uptake and markers of activation are indicated on right. (TIF) [file ppat.1009749.s007.tif]

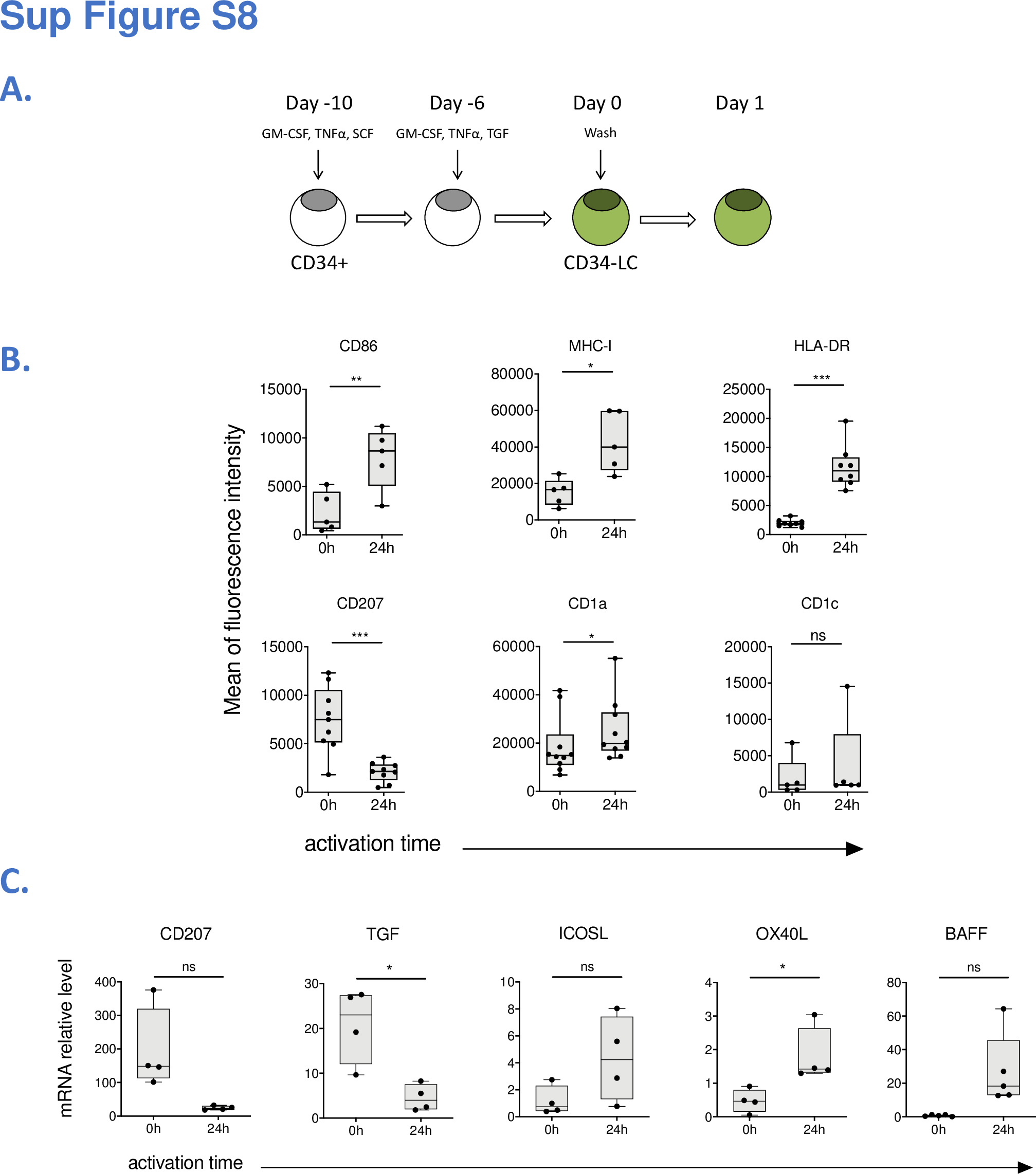

Supplement: S8 Fig — (A) Schematic of the procedure for maturating CD34-LC. Once differentiated, CD34-LC were cultured 24h without any cytokines to induce their maturation. (B) Expression of maturation markers of CD34-LC was monitored by flow cytometry (n = 9). (C) Total mRNAs from CD34-LC (n = 5) were isolated and real-time RT-qPCR of LC transcripts was performed as in Fig 5. Statistics were obtained using the non-parametric Mann-Whitney test (*, P < 0.05; **, P < 0.01; ***, P < 0.001; ns, non-significant). (TIF) [file ppat.1009749.s008.tif]

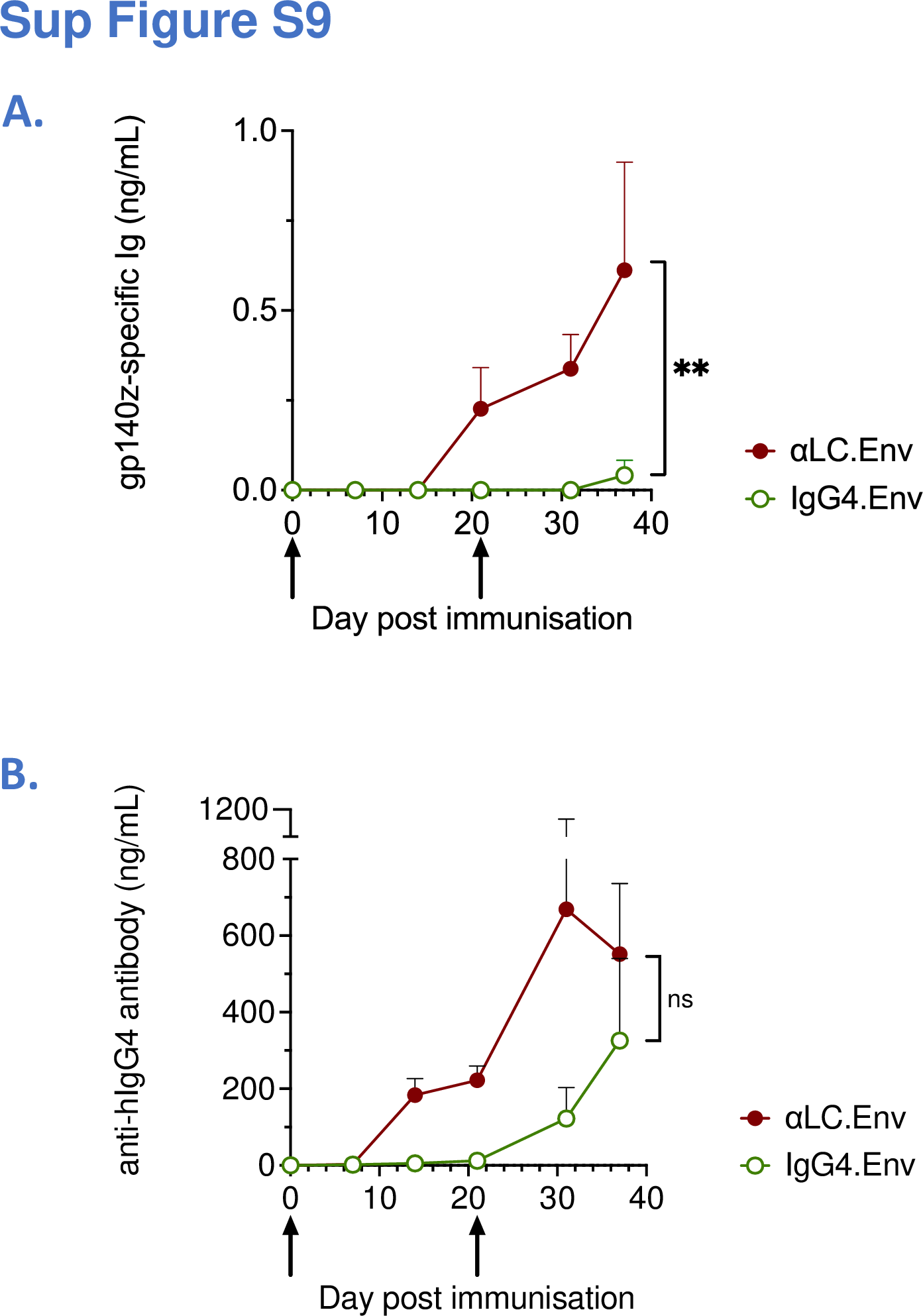

Supplement: S9 Fig — HuLangerin-DTR mice were immunized IP with 1 μg of αLC.Env (plain circles) or 1 μg of IgG4.Env (open circles), or with vehicle only (cyclo-dextrin buffer). (A) Env-specific antibody titers were measured by ELISA at day 14, 21, 31 and 38 post-immunization, using the 10.1074 antibody as reference control. Mean (± SEM) concentrations of each group (n = 3) are indicated. (B) Same as A, measuring anti-hIgG4 specific antibody amounts. Statistics were obtained using the Holm-Sidak’s multiple comparisons test (**, P < 0.01; ns, non-significant). Arrows indicate dates of injection. (TIF) [file ppat.1009749.s009.tif]

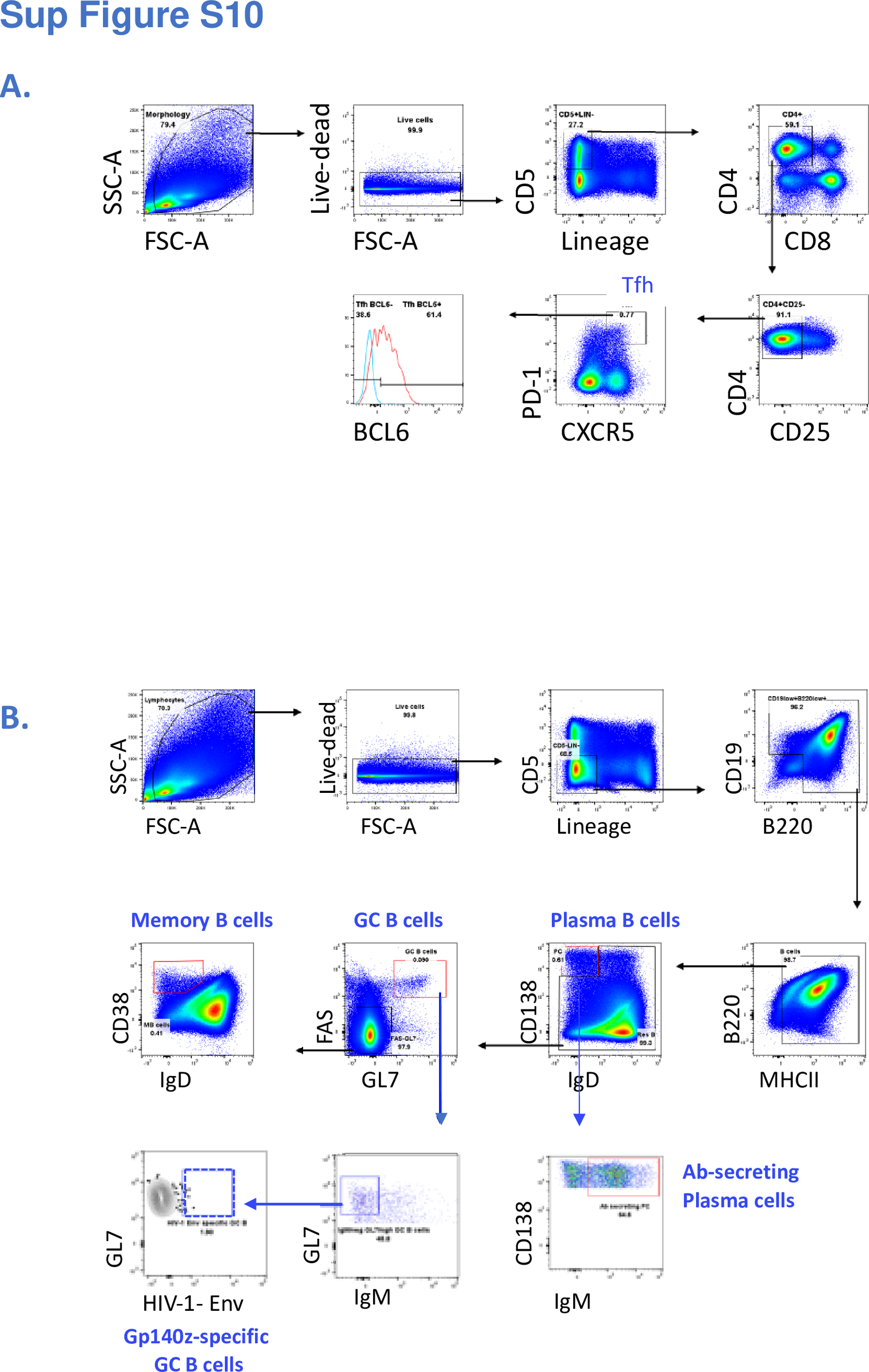

Supplement: S10 Fig — Single cells suspension of mice spleen and dLN were stained. (A) FACS-analysis and gating strategy of Tfh cells populations (spleen). Cells were gated on singlets, live and CD5+ Lineage- subsets. CD4+ CD8- T cells were separated in CD25- and analyzed for the co-expression of CXCR5+ PD-1+, characterizing Tfh cells. Tfh cells were checked for the expression of Bcl-6 (B) FACS-analysis and gating strategy of B cells populations (spleen). Debris, double and dead cells were excluded, and B cells were analyzed from CD5- Lineage- subset (CD11b- NK1.1-). Cells expressing low or positive values for CD19 and B220 were gated on MHC-II+ and then Plasma cells were identified by high expression of CD138 and null IgD. From those cells, the ones expressing IgM were identified as antibody-secreting Plasma cells. After exclusion of Plasma cells, GC B cells were gated on FAS+ GL7+. IgM+ B cells were excluded and IgM- GC B were analyzed for HIV-1.Env trimer specificity. Non-Plasma cells, non- GC B cells FAS- GL7- population was analyzed for CD38 and IgD expression. Memory B cells were defined as CD38+ IgD-. (TIF) [file ppat.1009749.s010.tif]

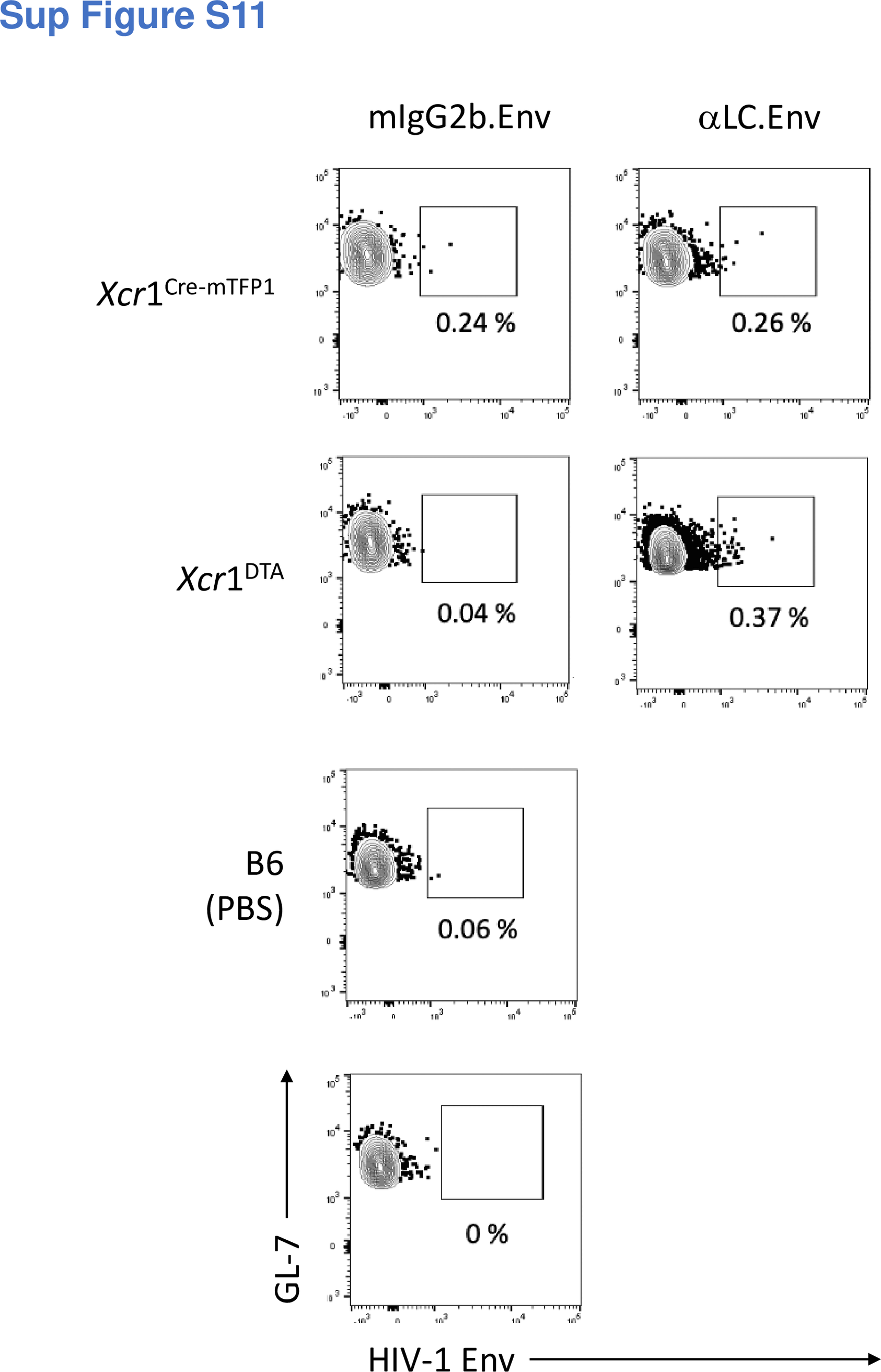

Supplement: S11 Fig — To identify GC B cells specific to HIV-1.Env protein, an in-house trimer of gp140z.biotin was made. Splenocytes were incubated previously with the trimer, washed and then stained for surface B-subsets specific antibodies. Debris, double and dead cells were excluded, and B cells were analyzed from CD5- Lineage- subset. B cells were defined as MHC-II+ low or positive CD19 and B220 expressing cells. After exclusion of plasma cells (CD138++ IgD-), GC B cells were identified on FAS+ GL7+ gate. GC B cells expressing IgM were excluded and IgM- GC B were analyzed for HIV-1.Env trimer specificity. Plots show frequency of parents for Env-specific GC B cells of one representative mouse per group. (TIF) [file ppat.1009749.s011.tif]

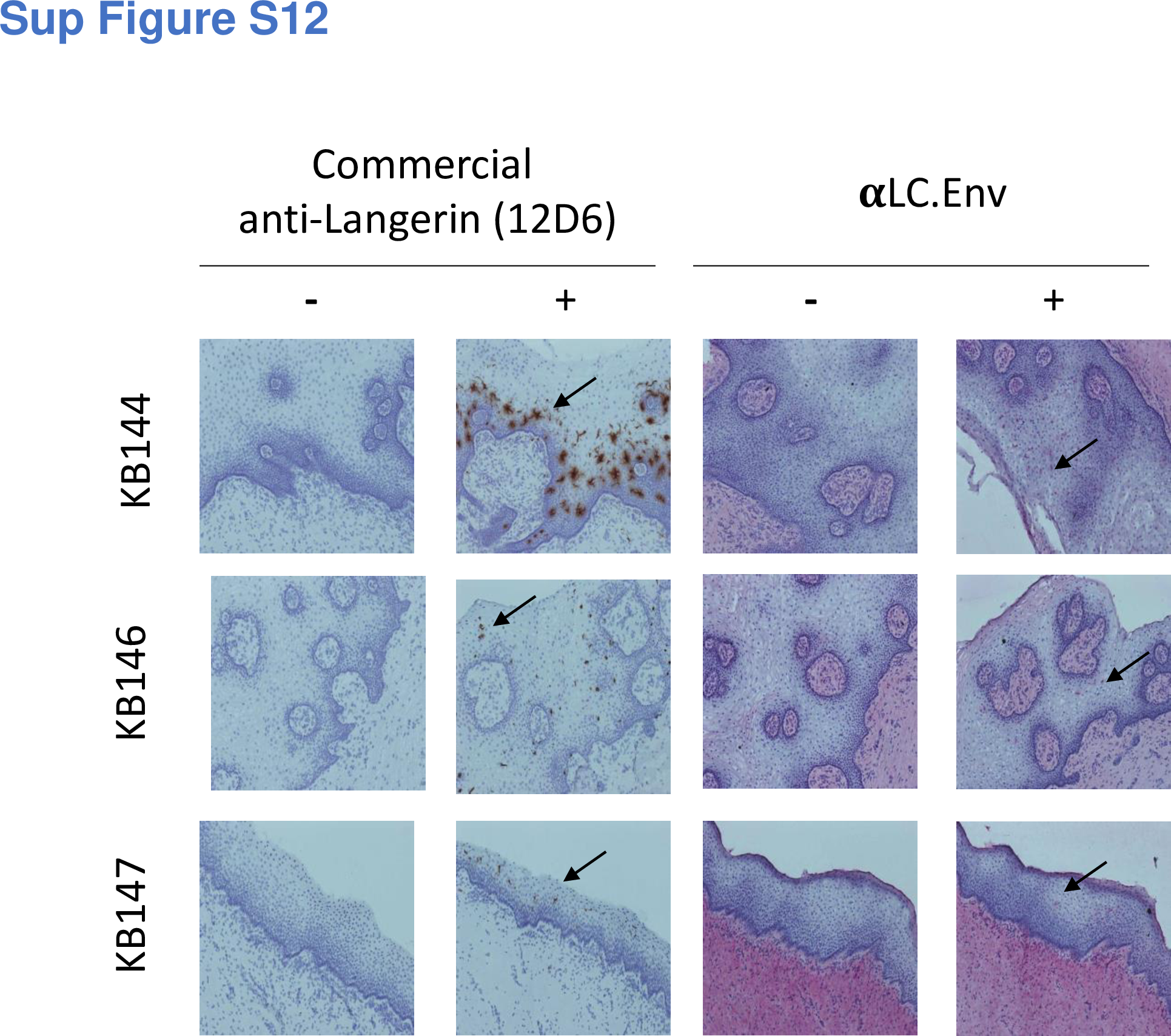

Supplement: S12 Fig — Langerin expressing cells of the vaginal mucosa were stained with (+) or without (-) a commercial anti-Langerin mAb (12D6) and revealed by peroxidase (brown). Concomitantly, additional sections were treated with αLC.Env and revealed by alkaline phosphatase (red). Langerin expressing cells appear located within the epithelium and cells binding αLC.Env are distributed in the same way. Arrows show examples of stained cells. Three representative donors are depicted. (magnification x10). (TIF) [file ppat.1009749.s012.tif]
