# Supplementary material for: Targeting human langerin promotes HIV-1 specific humoral immune responses
Source: PLoS Pathog. 2021 Jul 29;17(7):e1009749. doi: 10.1371/journal.ppat.1009749 (PMC8354475; doi:10.1371/journal.ppat.1009749)
Supplement: S1 Text — (DOCX) [file ppat.1009749.s013.docx]

**Supplemental Methods**

**Mice and immunization schemes**

HuLangerin-DTR mice, knock in for skin specific LC, have been previously described [1]. All animals were handled in strict accordance with good animal practice and complied with local animal experimentation and ethics committee guidelines. The Baylor Institutional Care and Use Committee approved all mouse protocols. Mice were a mixture of male and female of 8 weeks average age. Mice were immunized with 1μg equivalent of Env antigen intraperitoneally (ip) at day 0 and 21, without any adjuvant. Blood draw were collected as indicated in **Fig S9**.

**FACS-based binding assay**

Primary skin LC or CD34-LC were incubated in PBS 0.5% BSA with 3-fold serial dilutions of αLC.Env or control mAbs for 30 minutes on ice (30 nM to 0.3 nM). After washing, cells were stained with an anti-human IgG-AF647 (Biolegend) and phenotypical markers (CD45, HLA-DR, CD11c, CD207, CD1a) for 30 minutes on ice. Fixation of the tested mAbs was then assessed by flow cytometry.

**Internalization of the Langerin receptor**

CD34-LC were incubated overnight at 37°C with 10nM of αLC.Env or control mAbs. Either extracellular (extra) or extracellular and intracellular (total) CD207 staining was performed and the internalization of the Langerin receptor was calculated as followed: ((total %CD207) – (extra %CD207)) / (total %CD207). Extracellular phenotypical staining was added at the same time (CD45, HLA-DR and CD1a).

**Immunohistofluorescent staining of human skin explant**

Skin samples of three donors were frozen in OCT (Optimal Cutting Temperature, CML). Six μm thick sections were prepared using a cryotome. Samples were fixed using acetone and saturation was performed using gelatin from cold water fish skin (Sigma Aldrich). Then, samples were incubated with αLC.Env or control mAbs and the commercial mouse anti-Langerin (12D6, Abcam) overnight at 4°C. A goat anti-human IgG-Alexa Fluor 594 and a goat anti-mouse IgG-AF488 (polyclonal, Thermo Fisher) were added for 2 hours at room temperature. Nuclei were stained using DAPI. Finally, slide mounting was performed using VectaShield (Vector Laboratories).

**Immunohistochemical staining of human vaginal explant**

Samples were embedded in OCT compound and frozen. Eight μm thick sections were prepared, fixed 10 minutes with acetone and then treated with H_2_O_2_ and levamisole to inactivate endogenous peroxidase and phosphatase. Sections were incubated 15 minutes (RT) using a blocking buffer (2% Goat serum, 1% BSA, 0.1% Triton X-100, 0.05% Tween 20 in TBS), before adding 1h (RT) primary antibodies, either the commercial 12D6 (1:50; Abcam) or αLC.Env (20 μg/mL). No primary antibody was added to control background of the secondary antibody. Sections were washed in TBS 0.05% Tween 20. A commercial kit containing HRP polymer (Envision Plus Dual Link System HRP-DAB+, Dako) was used to reveal 12D6 staining, using DAB as substrate. For αLC.Env, a biotin-tagged anti-human Ab (Abcam) was added 30 minutes (1:4,000), followed by 20 minutes incubation with streptavidin-AP (1:200; Dako). A commercial detection kit (Fast Red substrate kit, Dako) was used to reveal the staining. Sections were counterstained with hematoxyline (30s) and mounted.

**Transcriptomic analysis by RT-qPCR**

LC were first analyzed for gene expression profiles by RT-qPCR as previously described [2]. Total RNA was isolated using Trizol Reagent (ThermoFisher) and 1 μg was reverse transcribed using ProtoScript II First Strand cDNA Synthesis Kit (New England Biolabs). SYBR Green PCR was performed with 2.5 ng of cDNA templates using commercial kit and Mx3005P QPCR System (Agilent) and GeneAmp 7300 Sequence Detection System (Applied Biosystems). The primer sequences for the PCR were as follows: GAPDH, 5’-TGG TCA TGA GTC CTT CCA CGA TA-3’ and 5’-CCC ATG TTC GTC ATG GGT GT-3’; CD207, 5’-CCA GGA ATT CAC ACC TGA CC-3’ and 5’-GCT TTA GTC AGG CCA ATC CA-3’; TGF_β1_, 5’-GGA GTT GTG CGG CAG TGG TTG-3’ and 5’-AGC TGA AGC AAT AGT TGG TGT CCA GG-3’; ICOSL, 5’-CAG GCT CTG CAG AAT GAC AC-3’ and 5’-TTT TCT CGC CGG TAC TGA CT-3’; OX40L, 5’-TGT CTG GGG ATG TGA TGC TT-3’ and 5’-TAG GCA GGA GGA TGA GCA TG-3’; BAFF, 5’-TGG TGA CTT TGT TTC GAT GTA TTC-3’ and 5’-GTT CAT CTC CTT CTT CCA GTT TTG-3’. Each sample was analyzed in triplicates, and the amounts of templates were estimated by linear regression against the known standard and normalized to internal control (GADPH).

**Illumina-based sequencing**

Second, mRNA libraries were sequenced on Illumina HiSeq/2500/V4 system. Total RNA was purified from cells using RNeasy Plus Micro Kit (Qiagen). RNAs were then quantified with Quant-iT RiboGreen RNA Assay Kit (ThermoFisher) before to be controlled for quality on the Bioanalyzer (Agilent). mRNA libraries were prepared by the NEBNext Single Cell/Low Input RNA Library Prep Kit (New England Biolabs). Libraries were sequenced on Illumina HiSeq 2500 V4 system. The sequencing depth mean was 40 million reads and the fragment length of 1X101bp. Sequencing quality control was checked using Sequence Analysis Viewer (SAV) and FastQ files were generated on Illumina BaseSpace. After trimming (QPhred score ≥ 25) with Bowtie 2- 2.2.5 software, reads were aligned to Human reference genome hg19 using STAR - 2.6.1 and quantified to annotation model hg19 - GENCODE Genes - release 19 using Partek E/M. Counts were normalized using counts per million (CPM + 0.01) method. Differentially expressed genes were identified using normalized read count as input to Gene-specific analysis (GSA) (Partek Flow). Only the genes with adjusted *P*-values (FDR) ≤ 0.05 and a fold change ≥1.5 were considered as differentially expressed. Functional enrichment analysis of differentially expressed genes was investigated using Ingenuity Pathway software.**References**

1. Flamar A-L, Zurawski SM, Scholz F, Gayet I, Ni L, Li X-H, et al. Noncovalent assembly of anti-dendritic cell antibodies and antigens for evoking immune responses in vitro and in vivo. J Immunol. 2012;189: 2645–55. doi:10.4049/jimmunol.1102390

2. Cardinaud S, Urrutia A, Rouers A, Coulon P-G, Kervevan J, Richetta C, et al. Triggering of TLR-3, -4, NOD2 and DC-SIGN reduces viral replication and increases T-cell activation capacity of HIV-infected human dendritic cells. Eur J Immunol. 2017; 818–829. doi:10.1002/eji.201646603
