# Supplementary material for: Targeting human langerin promotes HIV-1 specific humoral immune responses
Source: PLoS Pathog. 2021 Jul 29;17(7):e1009749. doi: 10.1371/journal.ppat.1009749 (PMC8354475; doi:10.1371/journal.ppat.1009749)
Supplement: Attachment — Submitted filename: Kervevan_response-reviewers.docx [file ppat.1009749.s014.docx]

Dear Editors

We thank the reviewers for their comments. We appreciate their suggestions to improve this manuscript and to highlight our results.

Reviewers pointed out strengths of our proposal. While we were testing targeting HIV Env to human LCs via anti-langerin antibody specifically induces Tfh cells and thence B cell memory and specific anti-env antibody secretion by antibody secreting cells, we used complementary approaches with *in vitro* functional assays of human primary LC T and B cells, transcriptomic and Luminex assays to investigate the mechanisms and finally *in vivo* immunization approaches to validate the immunogenicity of our vaccine candidate. Many evidence of the efficacy of DC-targeting vaccine against infection diseases and notably emerging diseases have been published during the last decade. Yet, few studies have been conducted to decipher the immunological function of LC and the impact of targeting this specific “DC” subset. In the context of HIV vaccine, reviewers highlighted the importance to develop new concepts, strikingly for improving antibody responses. Our study aimed to establish for the first time a proof-of-concept of the advantage of targeting LC for any further clinical development in humans.

Improvements within either experiments (and control assay), but also within the way to present / interpret our results have been made to respond to weaknesses identified by the Reviewers.

Globally:

1. We performed additional *in vitro* cultures of CD34-LC and epidermal LC with appropriate controls.
2. We performed additional immunization studies, using a different animal model (*Xcr*1^DTA^ mice developed by S Henri and coll. [1]) and a modified vaccine adapted to our mouse studies. We took the opportunity of these experiments to test the subcutaneous (sc) route of immunization and responses in draining lymph node (dLN). Our data demonstrate the advantage of the LC targeting for the induction of Tfh bcl6^+^ cells, GC formation and anti-Env specific B cell responses.
3. The manuscript was also reorganized i) to exclude non-essential results from figures, ii) to better refer to other studies overlapping our investigations and iii) to improve the reading.

Therefore, new collaborators have been involved in this project to perform additional experiments. Adele Hammoudi (PhD student fellow) worked on *in vitro* experiments with human LC, whereas Juliane Sousa Lonza (post-doctorate fellow), Sandrine Henri and Véronique Godot conceptualized and performed *in vivo* immunization assays. For note, these additional *in vivo* procedures have been submitted and approved by the Ethical Committee of Marseille (France).

Here are our responses and corrections to the specific points raised by each reviewer.

**Reviewer #1:**

**Summary**

1. **Kervevan and colleagues have fused HIV-Env to a langerin antibody (anti-Lang-env) and investigated its ability to induce an immune response via LCs. Firstly, they show in vivo (using mice expressing human langerin) that anti-Lang-env leads to robust production of IgG4 env specific antibodies and a high frequency env specific B cells that express germinal centre markers. They next targeted anti-Lang-env to human cord blood derived CD34-LCs and showed that autologous (cord blood?) naïve CD4+ T cells were differentiated into Tfh-like cells.**

The autologous and absence of cytokine is an important piece of our approach. Although the technique for culturing CD34-LC with naïve CD4+ T cells was already published (Bouteau et al. Front Immunol 2019)[2], we now indicate that autologous naïve CD4 T cells were isolated in the same cord blood donor as for CD34+ HSC.

- Please see lines 153 - 155:

*We first confirmed by staining of skin explant or dissociated epidermal cells that αLC.Env was binding Langerin^+^ cells in a dose-dependent manner, whereas no binding of IgG4.Env was observed (****Sup Figure S3A-B****).*

2. **When mixed with autologous B cells this led to the generation a very small (but significant) percentage of memory B cells some of which were of a CD21+ resting memory phenotype. Treatment of CD34-LC with also led to production of IgG2 and 4 (not IgG1 and 3) in this assay and then anti-env antibodies from B cells from allogeneic HIV+ donors apparently providing evidence of a functional interaction between naïve B cells and co-cultured autologous CD34-LC. They also investigated the costimulatory receptors and the array of cytokines secreted by CD34-LCs after ant-Lang-env treatment and showed some were consistent with Tfh differentiation and B cell function. Finally, they show in primary human skin LCs that similar cytokine expression profiles were seen.**

**Although we already know the approach (i.e. using anti-LC antibodies) works – these authors showed this in Ref#16 – I believe with major revisions this manuscript is of interest to the readers of PLoS Pathogens.**

As mentioned above, few publications focused on the targeting of Langerhans cells. Epaulard et al. (J. Immunol., 2014 [3]) tested our anti-Langerin mAb associated with the HIV-1 Gag protein. Whilst their results demonstrated in Non-Human Primates the advantage of targeting LC for inducing anti-HIV antibody responses, no evidence of the same functional response in humans was established. In addition, we developed in the present study an anti-Langerin vaccine associated with the Env protein with the perspective of developing a prophylactic vaccine. The publication mentioned (Bouteau A. *et al.,* Front Immunol. 2019 [2]) was geared to demonstrate the functional advantage of LC versus dermal DC at inducing antibody responses but the response to a vaccine antigen was not addressed.

3. **The underlying hypothesis is that targeting HIV env to human LCs via anti-langerin antibody specifically induces Tfh cells and thence B cell memory and specific anti-env antibody secretion by antibody secreting cells. They have used a complimentary mix of transgenic mouse, model human cord blood derived CD34-LCs and authentic human LCs and B cell from HIV+ patients (under allogeneic stimulation) to try to test this hypothesis Although these are valiant attempts the interpretation of the experiments falls short of proof of the hypothesis because of the lack of some controls, especially in the models systems.**

**Human CD34-LCs are very different to bona fide LCs by transcriptomics and HIV binding C-type lectin expression, overlapping with MDDCs and probably do not exist in vivo, at least not in stratified squamous epithelium. Thus, they need to show that Tfh are only induced by langerin and not other C-type lectin stimulation in these cells, confirm their findings with Tfh induction with authentic epidermal LCs and that other tissue DCs (preferably from anogenital epithelium or skin) do not have these properties. Indeed, we already know that skin DC (incl LC) drive TFH Ref#15**[4] **and GC responses Ref#12**[5]**. As env binds langerin directly then env itself (+/- conjugated to IgG4) should be compared to anti-langerin in all the above experiments to show any added benefit of anti-langerin conjugation.**

Previous studies have shown that targeting through different lectins or scavengers receptors expressed by DC drives different immune responses (Li et al JEM 2012; Zurawski et al J Virol 2017)[6,7]. It is now accepted that the choice of the targeted receptor will stimulate either different DC subsets exhibiting functional plasticity or intrinsic immune responses of the targeted DC, resulting of both the processing of the targeted antigen and adjuvant potency of the targeting vehicle (Yin et al Ebiosciences 2016)[8]. Therefore, we agree that stimulation through another receptor expressed by LC is an important question raised by our study.

Nevertheless, our study was not geared to establish a significant advantage of the Langerin targeting compared to another lectin receptor. The rational was to target Langerin which is one the main markers expressed by the Langerhans cells, and thus to ascertain the responses driven by this subset. We also included in all experiment schemes the non-targeting control (IgG4.Env) and an irrelevant αCD40.Env vaccine control. The immunogenicity of the αCD40.Env vaccine has been demonstrated (Zurawski et al J Virol 2017)[7] and motivated our choice. We added complementary experiments that are now presented in **Sup Fig S5**. CD34-LC were treated with αLC.Env, IgG4.Env and αCD40.Env as before but we also used: 1) the αDCIR.Env developed in-house (unpublished) and; 2) the αLC.Gag (Epaulard et al., J Immul 2014)[3]. In contrast to the αLC.Env, the Tfh cells induction was not changed by these treatments, at least in terms of cells number and expression of main Tfh markers (CXCR5, PD-1, ICOS, OX40L). While testing IgG production of memory B cells from HIV^+^ donors, we observed an advantage of the αLC.Env, which was significant as compared to the IgG4.Env, αCD40.Env and αDCIR.Env treatments.

- Please see new Sup figure 5
- Please see lines 190-194:

*A dose dependency between the amount of vaccine loaded on CD34-LC and the production of IgG was observed (****Sup Figure S5A****). This IgG production was not modulated neither when CD34-LC were treated with in-house developed vaccine targeting the HIV-1 Gag protein to the human Langerin, nor when targeting the HIV-1 envelop to another lectin receptor (DCIR) (****Sup Figure S5B****).*

Although further studies would be needed to study the consequences of activation of other lectin receptors expressed at the surface of LC, we believe that these investigations are out of the scope of the present study. As shown in **Figure** 1, several experiments have been performed using skin isolated LC showing consistent results with CD34-LC (**Figure 2**).

Finally, the Reviewer proposed to study isolated from ano-genital epithelium. As published by others [9], the very low frequency of Langerin-expressing cells in these epithelium makes these experiments very difficult. Moreover, these samplings require an IRB approval which was impossible to obtain in France during the last months in the period of COVID-19 pandemics.

4. **The mouse immunization experiments were intraperitoneal so local draining lymph nodes would be unlikely to contain LCs requiring more distal targeting to skin draining ing lymph nodes. As they mention in discussion mucosal immunization would drain to these lymph nodes so there a comparison of the need for env to be anti-langerin conjugated becomes more cogent.**

We agree that intraperitoneal (ip) is not the best approach to target skin LC and investigate responses in lymph nodes. However, in a previous study, using this route of immunization we demonstrated the importance of targeting Langerin expressed on the surface of skin LC to induce antibody responses (Bouteau et al Front Immunol 2019)[2]. To better address reviewer’s comment, we decided to further investigate immune responses generated by skin LC in another mouse model (X*cr*1^DTA^ mice) in which dermal DC (cDC1) expressing the Langerin receptor are depleted [1] and only LC express the Langerin receptor. We show, in **Figure 5** of the revised version that sc immunization with anti-Langerin vaccine drives a significant Tfh and Env specific responses, notably in dLN. Of note, the immunization through the vagina is currently under investigation by the VRI and will be the topics of another publication.

- Please see new figure 5
- Please see new paragraph # 4 “*Targeting HIV-1 Env in vivo to LC enhances antigen-specific B cell responses”.*

**Major issues**

1. **Model LCs derived from CD34+ cord blood monocytes were used. These are very different from bona fide LCs which are derived from the yolk sack. The authors should confirm with authentic tissue LCs in at least some parameters investigated in Figures 2 and 3, as mentioned above. Env and anti-langerin should be directly compared in all in vitro experiments**

Today, few experimental models have been published to differentiate progenitor cells into LC (Caux et al Blood 1997; Ganor Y AIDS 2008; Martínez-Cingolani C Blood 2014)[10–12]. We compared the differentiation of CD14^+^ or BDCA-1^+^ DC from circulating blood [11] versus CD34^+^ cells from cord blood [10]. It appears that the method we establish and that is based on Caux *et al.* allows the best differentiation of LC with high level of expression of CD207, CD1a and full expression of myeloid markers (CD45, HLA-DR, CD11c, CD1c). Representative staining are depicted in **Sup Fig 3**. However, we do agree with the reviewer that CD34-LC remain an *in vitro* model not reflecting full properties of primary skin LC. Therefore, to better answer to reviewer’s comment, we also tested primary skin LC for the activation of allogenic naïve B cells and we show that, under these conditions, there is a significant production of IgG when targeting freshly isolated epidermal LC (see **Figure 1C**). Moreover, transcriptomic analysis of maturating CD34-LC versus LC (now presented in **Sup Figure 7 and 8**) strongly suggest that coculture experiments of CD34-LC reflect functional properties of primary skin LC responses.

- Please see new figure 1 and Sup Figure S3, S7 and S8
- Please see lines 157-159:

*The treatment of epidermal LC with the αLC.Env vaccine construct significantly increased the production of total IgG (P < 0.01 and P < 0.05, as compared to the untreated or IgG4.Env non-targeted skin LC; respectively) (****Figure 1C****).*

- Please see lines 162-164:

*Whilst CD34-LC were phenotyped by the typical markers of LC, the αLC.Env construct was binding to CD34-LC in a dose-dependent manner and was shown to be rapidly internalized (****Sup Figure S3C-E****).*

- Please see lines 230-233:

*Strikingly, while skin LC were maturating, expression of ICOSL, OX40L and BAFF was upregulated, confirming that skin LC exhibit pro-Tfh functional makers during their migration (****Sup Figure S7A-B****). Of note, the same gene modulation was observed when maturing CD34-LC (****Sup Figure S8****).*

2. **The results in supplemental Figures 4 and 5 are important and should be a main Figures.**

We agree that these previous Sup Figures S4 and S5 are important since the Ms is geared to demonstrate before all cellular and molecular impacts of the αLC.Env. These figures are now depicted in **Figures 1B and 4** in the revised manuscript. Data from CD34-LC have been kept as Sup Fig S6 and S8 as comparison with skin LC.

- Please see new figure 1, 4 and Sup Fig S6 and S8

3. **The use of allogeneic HIV+ B cells in figure 3 is very artificial. Could this be augmented by testing ubiquitous antigens such as tetanus toxoid in an autologous system?**

We do not have the opportunity to get naïve B cell or memory B cells from HIV^+^ donors and skin explant from the same donor which requires IRB approval to get skin biopsies. Hence, we could not perform any autologous coculture with primary skin LC. The use of TT or ubiquitous antigen in an autologous system would definitely provide interesting evidence of the functional response of targeted LC and T/B responses. Nevertheless, we believe that it would be a completely different model of antigen, not reflecting anti-HIV responses driven by targeted LC. Moreover, doing these experiments would also require to design and produce new αLC fused to TT protein. These TT LC-targeting constructs are not available for the moment and the production of these vaccines are not in the agenda of our lab but could be cloned, produced and tested in the future.

4. **Why is there a difference in IgG subtype stimulation (3 and 4 vs 2 and 4) in figures 2 and 3.**

We honestly agree that we did not have any clear explanation of this discrepancy. We thank the Reviewer for raising this point, forcing us to reconsider these data. We decided to remove these data from the present Ms and to deeply investigate this question in the future.

5. **Much of the data in Figure 5 is unremarkable. As migratory LCs undergo maturation it is really no surprise at all that they cluster differently to immature cells isolated by trypsin digestion. Significant differences in clustering between mature and immature cells has been well demonstrated (e.g. PMID 23183897**[13]**). Genes associated with T cell stimulatory pathways are known to be specifically upregulated by APCs that spontaneously migrate out of dermis and epidermis as has already been well characterised in the literature (e.g. PMID: 28270408**[14]**, 17082627**[15]**). These and/or other similar studies should be referenced.**

We fully agree that differences in clustering between mature and immature LC have been well documented. Our primary goal was to confirm in our hands maturation/migration of skin LC induced expression of maturation markers but also the expression of molecules that may be responsible of the immunological functions of LC we observed. This figure is now presented in supplementary data (**Figure S7**) and we modified the text to include suggested references.

- Please see Sup Figure S7
- Please see lines 230-237:

*Strikingly, while skin LC were maturating, expression of ICOSL, OX40L and BAFF was upregulated, confirming that skin LC exhibit pro-Tfh functional makers during their migration (****Sup Figure S7A-B****). Of note, the same gene modulation was observed when maturing CD34-LC (****Sup Figure S8****). We then compared the transcriptional profile changes in maturing skin LC and investigated messengers for pro Tfh/B cell functions. Principal component analysis (PCA) distinctly showed two different clusters (****Sup Figure S7C****), underscoring a particular transcriptomic signature after migration, as already mentioned in another previous studies* [16]*.*

6. **Line 258 and 259: The authors state that langerin is exclusively expressed by LCs in humans. This is not correct. Some dermal cDC2 also express langerin (PMID: 25516751)**[17] **and recently anogenital langerin expressing epidermal DCs have been identified and characterized (PMID: 31227717)**[16]**. Indeed, epidermal DCs were shown to be far more efficient at HIV uptake and productive infection and transfer to CD4 T cells than LCs. In fact, by using CD1c to gate LCs the authors were probably selecting for epidermal DCs rather than (or certainly as well as) LCs (See PMID: 31227717 Figure 1A)**[16]**. The role of other human langerin expressing cells needs a detailed discussion as this envelope antibody may well be taken up by these langerin expressing cDC2 populations rather than LCs. Ideally the authors should compare epidermal CD11c+ cells to LCs**

We agree that we oversimplified the complexity of DC subsets present in dermal and epidermal layers. It is now established by recent transcriptomic analysis that other DC subsets express the Langerin receptor both in mice and humans. Indeed, Andrew Harman and coll. reported distinct epidermal mononuclear phagocyte populations (MNP), namely LC, CD11c^+^ DC and CD33^low^ MNP. While CD11c^+^ DC are also present in the anogenital tissues and actively participate to the transmission of HIV-1, CD33^low^ cells share LC transcriptomic profiles but do not seem to perform any antigen presentation. We performed FACS staining of trypsin-dissociated epidermal cells and confirmed the presence of these different mononuclear phagocytic cell subsets (illustrated in **Sup Fig S1**), although we did not have the opportunity to test the CD33 expression. The presence of any of these cell subsets in our experiments is indeed a crucial question now discussed in the Ms. However, in our case, sorted LC were HLA-DR ^int to^ ^high^, CD45^+^ together with CD1a^very high^. All cells were CD207^hi^. To us, this combination of markers partially excludes the presence of CD11c DC (CD207^low^) or the so-called CD33^low^ MNP subset (HLA-DR^low^) within sorted skin LCs. Therefore, we are confident that we isolated true LCs from epidermis. Nonetheless, we agree that the presence and the function of Langerin^low or high^ epidermal DC need to be taken into accounts when targeting the Langerin receptor. This part has been discussed as follow:

- Please see lines 307-326:

*Today, it is established that skin dendritic cells are heterogenous, can be distinguishable by surface markers and have distinct functional specializations* [5,13,18]*. By instance, CD33^low^ populations are transcriptionally similar to LC. They do not function as APC and are not targeted by HIV, whereas CD11c^+^ epidermal DC can transmit the virus* [19]*. Enzymatic digestions and procedures for isolating skin immune cells impacts on the detection of surface markers and thus on each DC subpopulation (e.g. CD11c, CD1c)* [14,19]*. Langerin-expressing DC are found in human tissues and are distinct from LC* [17]*. These cells also present in mucosa are prone to HIV-1 and to transfer the virus to the CD4^+^ T cells populations* [20]*. Also, the cDC1 subset in mouse dermis express Langerin, and presents equivalent functions with dermic human dendritic cells subsets* [21]*. Therefore, one cannot exclude in vivo that Langerin^+^ cDC could capture the αLC.Env vaccine or that antigen captured by LC could be transferred to another DC subset, as observed for HIV-1 particles* [22]*. Testing the targeting antigens to Langerin would require a functional comparison of other DC subsets present in the epidermis (as well as in the dermis), besides “truly” LC, and would constitute major steps for a better knowledge of the skin immunobiology and efficient development of vaccine. Our in vivo studies point to the generation of a more specific GC reaction in the SLO of mice when LC are targeted, especially in the absence of cDC1 (Xcr1^DTA^) suggesting that LC might be the most prone subset to sharpen the specific humoral immune response after vaccination. On the other side, we have presently evidence that targeting HIV-1 Env to the human Langerin receptor potentializes antigen-specific B cells responses in vitro.*

**Minor issues**

1. **There are numerous minor grammatical errors throughout the manuscript that need addressing.**

We thank the Reviewer for underlining this issue and our US collaborators helped us for a better writing.

2. **Line 128 – 131: Please provide a reference for this recent work (I assume ref 16).**

Indeed, the reference 16[2] was missing and is now provided.

- Please see lines 132-135

*We recently demonstrated that delivering a foreign antigen to steady state LC versus cDC1 through the same mouse Langerin receptor led to, respectively, strong versus minimal humoral immune responses* [2]*.*

3. **Please make sure to always use the term CD34 LC (rather than LC) when referring to model CD34 monocyte derived LCs (including in all titles).**

We modified the Ms and CD34-LC are now clearly named in the Ms as well as in Figures.

4. **LC isolation. I would recommend the use of collagenase IV rather than trypsin for LC isolation as leads to the isolation of cells with intact cell surface markers and with greater viability. Indeed, trypsin has been shown to cleave CD1c which the authors use to gate on LCs (PMID: 28270408)**[14]**. This could also influence differences in direct env vs anti-langerin-env targeting and stimulation of LCs**

We would like to thank the reviewer for this suggestion. The use of trypsin versus type IV collagenase is critical for the expression of some surface markers when isolating epidermal but also dermal cells. While we were isolating epidermal LC, we followed a protocol established by T Geijtenbeek and coll., and that we already experienced in a previous publication [22]. A critical step of this protocol is to do a short-time trypsin treatment (0.05% (w/v), 20 min at 37°C) to avoid any undesirable digestion of surface receptors. Bertram *et al.* reported that CD11c population cannot be distinguished after trypsin treatment of the epidermis [19]. They also reported a significant vanishing of the CD1c marker within the CD1a subset [14]. This team still used a 10 fold highest amount of trypsin (0.5%, w/v) for 30 to 120 min [14]. Differences in methods probably gave us the opportunity to appropriately gate on LC. Although difficult to estimate, we agree that a random part of epidermal LC was probably not isolated in our hands. Future studies including treatments with collagenase IV will be investigated to test whether it might improve present results.

- Please see discussion lines 307-326 (mentioned in point #4 major issues)

**Reviewer #2**:

**Summary**

**The authors report on a vaccine strategy in which antigen is fused to anti-langerin antibody. Injection of the recombinant fusion protein is thought to induce B cells to produce antibodies against the antigen. The strategy is also supposed to cause LCs to induce the differentiation of germinal center T follicular helper cells.**

**This is an interesting and innovative approach for introducing antigen into LCs that is worth exploring. The idea is that LCs may be better than DCs at promoting Tfh cell activation in GCs. The authors have done a considerable body of work using sophisticated cell culture methods. The experiments include relevant controls.**

**An overarching question about the study is whether this will be a valuable approach for an HIV vaccine. The problem with an HIV vaccine is not the inability to get an antibody response to antigen. The problem is inducing a broadly neutralizing response, or broadly acting CD8 T cells. bNAbs are produced at very low frequency in patients and only in rare individuals and no immunogens have been developed that induce a broadly neutralizing response. Targeting Gp140 to LCs is not going to solve this problem. Perhaps the approach will be useful for other specific antigens, at least if it could be shown that the immunization results in better antibodies than just injecting antigen. The study does not convincingly make that case.**

**A weakness with the experiments is that some of the effects are quite modest and some do not seem to be significant. Furthermore, the data do not show that the approach is any better than standard approaches of injecting purified antigen. Most of the data presented just show the antibody response to the antibody IgG4 carrier. Very little is done to look at the anti-gp140 antibody response.**

**An important consideration is whether the study is appropriate for PLOS Pathogens. It is not aimed at a pathogen but is an immunological report to look at Tfh differentiation and antigen targeting to LCs. It would more suited to an immunology journal, of which there are many.**

We would like to thank the referee for these comments. We do agree with the referee that one major goal of the prophylactic vaccine field is to induce bNAbs. However, the referee would also agree that, to date, this goal is not achieved with any vaccine candidate even tested in these 2/3 trials. Moreover, in the RV144 trial, the only trial showing a partial protection, vaccine effect was not associated with the generation of bNAbs in vaccinees. Nevertheless, our study is a proof-of-concept study aimed to decipher the mechanisms of the induction of immune responses by targeting antigens to Langerin. One point raised by the referee is the added value of targeting the antigen as compared to injection of the protein alone. To better answer to the reviewer’s concern, we have performed new experiments to address this point (please see below and the revised manuscript).

**Major issues**

1) **Fig. 1A. The figure shows the anti-IgG4 antibody response but does not show the anti-gp140 antibody response. A histogram needs to be added to show the anti-Gp140 antibody titer. The anti-IgG4 response is not what’s important, it's the anti-gp140 antibody that matters. Panel C does shows a time course of anti-Gp140, but that is not sufficient. If the approach is designed to induce antibody against the antigen linked to anti-langerin, then the titer of anti-Gp140 antibody is a central question.**

We agree that investigating responses against the IgG4 backbone has been already reported, although in a different mouse model [2]. In a new set of experiments using huLangerin-DTR mice, Ig responses against the gp140z were also observed when immunizing with anti-Langerin.gp140, indicating an advantage of targeting the langerin-expressing cells to stimulate a response against the vaccine antigen. Time courses of antibody responses against gp140z in huLangerin-DTR mice immunized with αLC.Env vs IgG4.Env are now shown as **Sup Figure S9**. Moreover, we performed new experiments with vaccine constructs in which the human IgG4 backbone of the antibody has been replaced by a mouse IgG2b, limiting thus the allogenic anti-vaccine responses observed in the previous mouse studies. We also used a mAb, which cross-reacts with the mouse Langerin, as previously reported by our team [23]. In addition, we decided to use the *Xcr*1^DTA^ mice in which dDC (cDC1) are depleted [1], pointing thus vaccine humoral responses activated by epidermal LC. Strikingly, we now demonstrate that subcutaneous (sc) immunization with anti-Langerin vaccine drives a significant Tfh and Env-specific GC B cell responses, notably in dLN. Of note, the immunization through the vagina is currently under investigation by the VRI and will be the topics of another publication.

- Please see Figure 5 and Sup Fig S9
- Please see new paragraph # 4 “*Targeting HIV-1 Env in vivo to LC enhances antigen-specific B cell responses”.*

2) **Fig. 1B (2B ?). The protocol involves adding anti-CD3/CD28 to the cocultures. The treatment is a strong T cell activator and one that would not be used in a vaccine. The vaccine needs to work in the absence of such a treatment. The authors need to demonstrate that the vaccine can stimulate a B cell response in the absence of global T cell activation.**

With respect of the referee, several *in vitro* test to evaluate specific responses use a coactivation system to amplify the expansion of specific cells (evaluation of *in vitro* cell producing cytokines “ICS” for example). However, we also performed, in a first approach, experiments without using CD3/CD28 stimulation to let naïve CD4^+^ T cells differentiate into Tfh. Results were reported in Bouteau et al.[2]. Nevertheless, the intensity CXCR5 / PD-1 phenotypic staining appeared quite dim, although the Bcl6 expression of this population was confirmed confirming the differentiation of Tfh cells. However, since the differentiation was confirmed although with a low percentage of differentiated Tfh cells (median 20%), we used a co-activation system to expand more dramatically the frequency of these cells to improve the differentiation and Ig production by cocultured B cells. The CD3/CD38 stimulation led to 75 to 90% of Tfh differentiated cells. Yet, *in vitro* models will never reflect the complexity of cells interactions occurring the lymph node. Our model was established to provide reproducible and significant results and thus get evidence of these cell interactions.

**Also, the size of the font of the text included in the flow plots needs to be increased for readability.**

We thank the Reviewer for noticing this issue. We worked to improve their readability of all flow plots.

- Please see Figures 1-2 and Sup Figure S1, S2, S3, S4, S10 and S11

3) **Fig. 1C. This is one of the few analyses of the anti-gp140 antibody but the data it is weak and could be interpreted to suggest that the approach is not a good one. The anti-gp140 antibody is about 1/500 of the anti-IgG4.**

As mentioned above, initial mouse studies are now reported in **Sup Figure 9**, whereas a novel immunization approach is now presented in the new **Figure 5** of the revised manuscript. The anti-gp140z antibody responses have been evaluated using a Luminex-based approach developed by Craig Fenwick and coll [24]. We now show a more that a log higher Ig response against the gp140z antigen when immunizing mice with LC-targeting Env mAb versus mIg2b non-targeting vehicle. Taken together, these animal studies provide new data demonstrating the added value of the LC targeting of the HIV Env antigen as compared to the non-targeted one. Last, additional FACS data of Tfh and B cells populations generated in the spleen and dLN stressed the improvement of the Tfh / B cell responses against the Env antigens when mice were immunized with a LC-targeting vehicle.

- Please see Figure 5 and Sup Fig S9
- Please see lines 268-271:

*Levels of Env-specific IgG in sera were measured by a Luminex-beads assay* [24]*. Strikingly, we observed more than one log difference of anti-Env IgG in mice immunized with αLC.Env vaccine as compared to the mIgG2b vaccine (****Figure 5A****)..*

- Please see new paragraph # 4 “*Targeting HIV-1 Env in vivo to LC enhances antigen-specific B cell responses”* for additional tests of Tfh and Env-specific GC B cell responses.

4) **Fig. 2A. The authors switch here to human cells. They need to make this more clear by stating it at the beginning of this section in Results.**

We agree that the rationale of our investigations from animal to *in vitro* models was not clearly stated. Therefore, we now propose to first introduce *in vitro* analysis of human cells (**Figures 1 to 4**) and then to present our investigations deciphering *in vivo* responses of the LC-targeting vaccine (**Figure 5**).

5) **Fig. 2D. The effects shown are small. There is no response for Ig A, M, G, G1, G2. The IgG3 is negligible and the IgG4 is very small. The findings seem to suggest that the approach is not good.**

As mentioned above (Reviewer #1, Major point 4), we reconsidered globally the impact of αLC.Env on the production of different subtypes of Ig by naïve and memory B cells. Ig production was limited and could be explained by 1) the limited number of B cells that can be added per well, and ii) the limited time of culture; 3) the very low frequency of cells capable to differentiate *in vitro* in this culture system without addition of large cocktail of cytokines. Although narrow, we clearly observed by ELISA a significant production of Ig with αLC.Env (**Figures 1C and 2C**). Due to the discrepancies in our IgG subtypes analysis, we decided to remove these data from the present Ms and to investigate this question later.

6) **Fig 3. Here the experiment uses HIV+ patient B cells. The text needs to make this more clear. The rationale is presumably to increase the number of B cell clones expressing anti-gp140 antibody. This needs to be more clearly stated. This vaccine is not a therapeutic vaccine. If it requires previously primed B cells, it is not going to work.**

We agree that HIV-1 Env-based vaccine are primarily geared to be tested in prophylactic clinical assay. Whilst modest, protection achieved in the RV144 trial was associated with the generation of anti-Env antibodies binding to the V1V2 region. Today, ongoing clinical trials intend to explore different strategies, relying on the induction of neutralizing antibodies or cellular immunity, which at term could be beneficial in a prophylactic setting. Our objective here was to show that targeting Env to Langerin, as compared to non-targeted antigen, induced the generation of Tfh cells *in vitro* and in a set of new *in vivo* experiments, GC B cells. Elicitation of Tfh cells in higher frequency with αLC targeting, is a first step for improving B cell differentiation and B cell responses. Of course, the differentiation of these cells *in vivo*, generation of somatic hypermutation and NAbs is definitely a more complex issue and would require testing these vaccines in more appropriate models or in human, which is beyond the goal of this proof of concept study.

In our study, we first demonstrated that targeted skin LCs induce naïve B cell differentiation into class-switched plasmablasts (**Figure 1**). In a previous work, H Joo *et al*. tested in a similar way the targeting of the LOX-1 receptor expressed by DCs and activation of naïve B cells [25]. Therefore, we decided to investigate activation of naïve B cells in a complete autologous system, without any cytokine and using for this, CD34-LC.

- The rationale of this approach is now stated on line 160-162:

*We then investigated the differentiation of naïve B cells in a complete autologous system. Therefore, we differentiated in vitro LC from CD34^+^ cord blood cells (CD34-LC), as previously described* [2] *(****Figure 2A****).*

To our knowledge, the *in vitro* modeling of B cell priming is limited by the repertoire of cultured naïve B cells and the limited reconstitution of the lymph node environment. Hence, the production of Env-specific Ig by primed B cannot be achieved. Consequently, we decided to use circulating memory B cells from HIV^+^ donors, to provide any evidence that targeted CD34-LC are able both to promote a T-dependent B cell activation but also to present the vaccine antigen and thus to promote Env-specific B cell responses, without any addition of cytokine (**Figure 3**).

- The rationale of this approach is now stated on line 182-186:

*The in vitro priming of HIV-Env B cells is limited by the frequency of circulating progenitors and the difficulty to reproduce the lymph node environment. Therefore, we decided to investigate whether the targeted human LC were able to stimulate the production of Env-specific antibodies by memory B cells. Autologous CD34-LC/Tfh cells were cultured with total blood B cells from allogenic HIV^+^ donors, without any additional cytokine (****Figure 3A****).*

7) **Fig. 3D. This is the only other panel that looks at the anti-gp140 response. It shows an increase in antibody to gp140 of about 2-fold over background. This seems to be weak. It does not state the titer of the anti-gp140 antibody.**

As mentioned above, the number of cultured B cells is limited for each donor and time of culture was conditioned by the survival of B cells. Long-term culture of B cells requests pre-stimulation of B cell and addition of cytokines as reported by our group and others [25,26]. These conditions will let us envisage to improve the amount of antibody present in culture supernatant. Yet, we were privileging a no cytokine treatment of cells and no anti-CD40 stimulation to better reveal the functionality of differentiated Tfh cells. At this step of the project, we do believe that the detected amounts of Env-specific antibodies are highly reproducible and statically significant. Note that the limited amount of supernatant does not allow to titer anti-gp140 Ab, albeit we concede that this result would also be informative. We believe also that the low frequency of HIV-specific B cell precursors, in culture condition without cocktail of cytokines improving B cell expansion and differentiation make very difficult to get large amounts of Env-specific IgG in the supernatants. Therefore, we performed additional immunizations of mice to complete our observations in *in vitro* experiments.

8) **Fig 4B. The cytokine response is difficult to judge from the figure. The legend at the right goes to >2-fold. There are only 2 cytokines that are increased (CXCL13 and IL2) and if these are less than 2-fold increases then the response is minimal. It is not possible to compare the color of the squares which appear to be gray with the colors on the legend.**

We thank the Reviewer for noticing these issues, both about the readiness of the figure and the interpretation of the results.

We worked to improve their readability of this initial Figure 4. Data of cocultures with skin LC are now presented in **Figure 4**. In **Figure 4A**, we reported amounts of each cytokine, depending on tested conditions: i) skin LC alone, ii) skin LC + naïve CD4^+^ T cells for 4 days, and iii) coculture with total B cells. Note that skin LC with B cells alone was not tested since B cells do not survive without differentiated Tfh cells. **Figure 4B** detailing IL-21 and APRIL highlights the production of these cytokines in the presence of Tfh and B cells. **Figure 4C** shows values of IL-18 and CXCL13, since the production of these cytokines were more than two times higher with αLC.Env treated skin LC than with untreated skin LC. These increases were higher than for Ig4.Env but still non-significant. These new analyses are now detailed in paragraph #3 “Targeting skin LC promotes pro-Tfh / B cell functions”, showing together with transcriptomic analyses that LC express markers and produce cytokines during maturation that can promote Tfh and B cell responses, and that αLC.Env treatment emphasizes these changes in terms of functional profile. Data were supported by investigations in CD34-LC showing similar profiles (see **Sup Figure S6**)

- Please see new Figure 4 and Sup Fig S6, S7 and S8.
- Please see new paragraph # 3 “Targeting skin LC promotes pro-Tfh / B cell functions*”.*

9) **Fig. 5. The figure is an experiment to look at the phenotype of LCs. It has nothing to do with the anti-langerin-gp140. It thus, does not fit in with the study. The authors refer to Fig. S4C, which does use the fusion protein. It would seem that this is the relevant experiment and should be in the main text, not the supplement.**

As mentioned above (Reviewer #1, point #5), we do agree that even though the expression of pro-Tfh/B cell markers observed during the maturation of CD34-LC and primary skin LC showed some evidence of the Pro-Tfh/ B cell functions, this result overlaps previous publications. Then, Fig 5 is now presented as **Sup Figure S7**, these data remaining informative to validate the model we developed. We also added pertinent references describing expression of maturation and functional markers of migrated LC. Impact of the vaccine on the pro-Tfh / B cell functions of LC is now depicted in **Figure 3** for skin LC and **Sup Figure S6** for CD34-LC.

- Please see new Figure 4 and Sup Fig S6, S7 and S8.
- Please see new paragraph # 3 “Targeting skin LC promotes pro-Tfh / B cell functions*”.*
- Please see lines 235-237

*Principal component analysis (PCA) distinctly showed two different clusters (****Sup Figure S7C****), underscoring a particular transcriptomic signature after migration, as already mentioned in another previous studies [16].*

10) **Fig. 1A. A histogram needs to be added to show the anti-Gp140 antibody titer. The anti-IgG4 response is not what’s important, it's the anti-gp140 antibody that matters.**

We agree that anti-IgG4 is anecdotic, and this result is now reported in **Sup Figure S9**. In fact, we decided to reconsider globally our immunization studies. Initial immunization experiments were performed in the HuLangerin-DTR mice and aimed to unravel humoral responses generated by the αLC.Env vaccine construct that was tested *in vitro* on human cells (depicted now in Figures 1 to 4). The time-course of quantifying anti-gp140z Ig showed a significant response when mice were immunized with langerin-targeting vehicles (depicted now in **Sup Figure S9**). Still, we agree that responses were low, rending impossible any titer quantification. Therefore, we modified the vaccine i) by using the 4C7 mAbs able to bind the mouse Langerin receptor (as previously published in Bouteau et al, Front Immunol 2019)[2], and ii) by replacing the human IgG4 backbone by a mouse IgG2b backbone, reducing thus any non-specific antibody response.

- Please see new Sup Fig S9 and Figure 5.
- Please see also reply to **Reviewer 1 (Point #4, page 6)**.

**Minor issues**

**Some of the fonts in the FACS plots are too small to be readable.**

We thank the Reviewer for pointing this issue. Previous and newly obtained flow plots have been worked to improve their readiness.

**Reviewer #3**:

**Summary**

**In this manuscript the authors describe the use of a novel and interesting reagent i.e an Ab specific to Langerin, a molecule expressed in skin DCs to target the HIV env protein. They show that targeting this molecule to DCs in mice will lead to the development of Abs to env. They dissect mechanistically the development of these env specific Abs. They show that naive T cells added to cultures of in vitro matured Langerhans cells will differentiate into Tfh cells that will provide help in these cultures to B cells from HIV + subjects. This help will lead to production of env specific Abs. They perform RNA seq experiments on these in vitro matured Tfh cells and show that these cells adopt some of the features (cell surface markers and cytokines generic to Tfh cells).**

**The authors of this manuscript have developed this reagent to address an important need i.e how to enhance the production of Abs to HIV env protein. Unfortunately the manuscript falls short of providing a real solution that can be used in the context of an HIV Cure or an HIV prevention .**

- **What is the rationale for targeting skin DCs; while skin DCs can prime B and T cells, skin is not the most prevalent mode of transmission; hence one weakness is the relevance of this reagent**
- **The authors do not show that these Abs can neutralize HIV or act as broadly neutralizing Abs or prevent HIV infection in SCID hu models of HIV infection or let alone in vitro**
- **The experiments are descriptive, and the authors fail to provide or more importantly validate any of the suggested mechanisms of action namely promoting Tfh differentiation**
- **The bioinformatic analysis can be significantly improved**

We do understand concerns raised by the Reviewer, and it is essential to strength these questions in the Ms.

The enormous diversity of HIV-1 is a significant impediment in selecting envelopes that can be suitable for designing vaccine immunogens. Native-like Env proteins, with by instance soluble trimeric forms, have been developed. While tremendous progresses have been made, very few reports showed their capacity to elicit neutralizing Abs in animal models. Various strategies have been proposed for selecting and generating the suitable antigen, with by instance the sequential use naturally occurring Env [27] or the use of Env sequences promoting the expansion of bNAbs germline precursors [28]. Albeit the selection and design of this antigen is crucial, the proper capture and presentation of this antigen in the germinal center has equal importance to promote the vaccine response. In this context, we do believe that properly targeting the antigen to functional skin APCs is of importance to stimulate to anti-HIV B-cell repertoire. The LC targeting proof-of-concept developed in the present Ms will ground innovative axes for developing HV vaccine, with by instance the opportunity to target best-in-class HIV-1 antigens to the skin LC and thus to efficiently promote the humoral responses within the dLN. This program has been already initiated by the VRI.

Targeting LC with the skin epidermis has been shown to help for GC reaction and priming of specific T and B cell responses [4,5]. It is obvious that improvement still need to be done to properly administer the vaccine with skin layers, and thus deliver the vaccine to the DC subsets. Hence, mAb specific of the DC receptor will definitely help for addressing the vaccine antigen to functional APCs, even at a minute amount. Systemic and mucosal responses to LC-targeting vaccines have been reported. As mentioned in our discussion, it might be possible in the future to target *in situ* Langerin-expressing cells present with the mucosa. Further investigations will be conducted to decipher whether targeting mucosal epidermal “LC” improves the local responses and thus prevent HIV-1 transmission at the site of entry. These investigations are not within the scope of the present study.

As previously discussed (**see Reviewer 2, point 7, page 17**), the development of HIV-1 vaccine is axed on a proper induction of Env-binding Ab and cellular responses. The induction of broad-spectrum neutralizing antibodies is one of the main avenue of research from an immunological point of view. Binding and non-neutralizing Ab are also of interest to promote HIV protective responses and reduce viral reservoir [29]. To our knowledge, generating *in vitro* neutralizing Ab of vaccine interest remains unrealistic and this could be envisaged only in animal models. The recent development of KI mouse model with a biased B cell repertoire towards the generation of bNabs producing cells is a tremendous tool to decipher whether vaccine antigens or vaccine strategy could promote protective responses [28]. They constitute a first step before moving towards NHP SIV or SHIV models. Taken together, we should not overwhelm our experimental approach and results as a first step before addressing more specific questions on Ab functionality in animal models.

**Major issues**

The authors should

1. **Show that these Abs can neutralize HIV infection in primary CD4 T cell models of HIV infection and in SCID hu mouse models of HIV infection.**

As mentioned above, we do believe that it would not be realistic to consider that *in vitro* culture of circulating B cells (naïve progenitors or HIV memory B cells) will specifically stimulate the B cell repertoire of NAbs and/or bNAbs. We concede that after a long-term culture of B cells with many rounds of stimulation, the amounts of Ab produced in the SN could be analyzed for their binding to Env (on infected CD4^+^ T cells by instance) or they capacity to neutralize HIV-1 cell to cell transmission. Yet, questions we address focus on early events of the LC/naïve T and B interactions and we performed without any artificial stimulations of the cells (no use of CD40L, no cytokines added). Regarding animal models, the knock-in mouse model established by W Schief, B Haynes and other labs would be genuine models to study whether the targeting vaccine can stimulate germline precursors of B cells secreting bNabs, as determined by another Env-derived antigens [28]. These experiments are ongoing but these specific models are beyond the scope of the present manuscript aimed to analyze *in vitro* and *in vivo* effects of targeted antigen versus non-targeted. We have performed a new set of experiments to address these points (please see below).

2. **Provide validated mechanisms that can support the hypothesis raised by the authors i.e Langerin targeted LCs can trigger the differentiation of naïve T cells to Tfh cells. In these experiments the authors should provide convincing evidence that the Tfh cells were not a contaminant of the original input . They should perform spiking experiments and titrate in Tfh as their positive control . They so do not have a benchmark to show what bonafide Tfh cells can do to enhance the production of env specific Abs.**

We do understand concerns raised by the Reviewer. We previously published that CD34-LC induce the differentiation of naïve CD4^+^ T cells into Tfh cells in a complete autologous system [2]. We now show that skin LC drive the differentiation of allogenic naïve T cells into Tfh cells in our culture system (**Figure 1**). Note that all sorted populations were controlled by Flow cytometry. No Tfh cells were detected within the purified naïve CD4^+^ T cells population (see **Figure S2**). To demonstrate the impact of αLC.Env, additional controls were added (e.g. αDCIR.Env) and dose-responses of loaded vaccine were tested (see **Sup Figure S5**). We could not detect any significant differences for the Tfh cells differentiation, at least by staining classical Tfh markers. Still, we observed a significant and reproducible impact on the production of IgG by cocultured either with naïve B cells (as presented in **Figure 1 and 2**, for skin LC and CD34-LC respectively), or total B cells (as presented in **Figure 3**). Together with the production of IL-21 detected in supernatant (**Figure 4**) and the fact that IgG production is not modulated without adding naïve CD4^+^ T cells, we have evidence of a B cell activation depending on the Tfh functions.

- Please see new Figures 1-4 and Sup Figure S2 and S5.
- Please see our complementary responses in **points 6 and 8 of Reviewer #2 (pages 16 and 18).**

3. **Adding increasing numbers of Langerin targeted LCs or of naïve cells to these cultures and show a dose dependent increase in Ab titers is an important control**

We agree that a dose dependent response of targeted LC is an important control. We could not optimally perform a large range of LC/T ratio, due to the limited number of cells. Yet, we assessed increasing doses of vaccine loaded on LC (using αLC.Env versus IgG4.Env) and analyzed T and B cells responses. Results are shown in **Sup Figure S5**. We show that the production of total Ig by allogenic B cells was dependent on the dose of loaded vaccine, with a higher production with αLC.Env.

- Please see new **Sup figure S5**
- Please see line 190-194:

*A dose dependency between the amount of vaccine loaded on CD34-LC and the production of IgG was observed (****Sup Figure S5A****). This IgG production was not modulated neither when CD34-LC were treated with in-house developed vaccine targeting the HIV-1 Gag protein to the human Langerin, nor when targeting the HIV-1 envelop to another lectin receptor (DCIR) (****Sup Figure S5B****).*

4. **The bioinformatic analysis should involve comparisons to bonafide Tfh signatures published in the literature. The analysis while suggestive are incomplete**

We reported expression of some markers that are upregulated during LC maturation and that might participate to the Tfh/B cell functional response. Thus, our descriptive transcriptomic analysis are now presented in **Sup Figure S7** and previous publications confirming our observations are now referenced.

- Please see new **Sup figure S7**
- Please see our complementary responses to **Reviewer #2** (**Point 10, Page 20).**

5. **The authors have identified several cytokines and cell surface markers generic to Tfh cells but they do not show that these molecules play a role in the enhancement of production of env specific Abs. They should perform blocking experiments.**

Experiments using various antibodies or products either blocking interactions between cell surface molecules or cytokines would be useful to demonstrate precisely mechanisms involved in LC/naïve T cell interactions or key molecules for the differentiation of Tfh cells. However, as already stated, the low frequency of LC available to perform coculture experiments did not allow to test various hypotheses. Our main goal was to demonstrate that targeting LC improved *in vitro* differentiation and generation of Tfh as compared to non-targeted capture of vaccine antigens.

- Please see our complementary responses and conclusions relative to the cytokine environments in **Point 9 of Reviewer #2 (Page 19).**

**References**

1.	Wohn C, Le Guen V, Voluzan O, Fiore F, Henri S, Malissen B. Absence of MHC class II on cDC1 dendritic cells triggers fatal autoimmunity to a cross-presented self-antigen. Sci Immunol. 2020;5. doi:10.1126/sciimmunol.aba1896

2.	Bouteau A, Kervevan J, Su Q, Zurawski SM, Contreras V, Dereuddre-Bosquet N, et al. DC subsets regulate humoral immune responses by supporting the differentiation of distinct TFH cells. Front Immunol. 2019;10: 1–15. doi:10.3389/fimmu.2019.01134

3.	Epaulard O, Adam L, Poux C, Zurawski G, Salabert N, Rosenbaum P, et al. Macrophage- and Neutrophil-Derived TNF- Instructs Skin Langerhans Cells To Prime Antiviral Immune Responses. J Immunol. 2014;193: 2416–2426. doi:10.4049/jimmunol.1303339

4.	Yao C, Zurawski SM, Jarrett ES, Chicoine B, Crabtree J, Peterson EJ, et al. Skin dendritic cells induce follicular helper T cells and protective humoral immune responses. J Allergy Clin Immunol. 2015;136: 1387-1397.e7. doi:10.1016/j.jaci.2015.04.001

5.	Levin C, Bonduelle O, Nuttens C, Primard C, Verrier B, Boissonnas A, et al. Critical Role for Skin-Derived Migratory DCs and Langerhans Cells in TFH and GC Responses after Intradermal Immunization. J Invest Dermatol. 2017;137: 1905–1913. doi:10.1016/j.jid.2017.04.016

6.	Li D, Romain G, Flamar A-L, Duluc D, Dullaers M, Li X-HX-H, et al. Targeting self- and foreign antigens to dendritic cells via DC-ASGPR generates IL-10-producing suppressive CD4+ T cells. J Exp Med. 2012;209: 109–21. doi:10.1084/jem.20110399

7.	Zurawski G, Shen X, Zurawski SM, Tomaras GD, Montefiori DC, Roederer M, et al. Superiority in Rhesus Macaques of Targeting HIV-1 Env gp140 to CD40 versus LOX-1 in Combination with Replication-Competent NYVAC-KC for Induction of Env-Specific Antibody and T Cell Responses. J Virol. 2017;91: JVI.01596-16. doi:10.1128/jvi.01596-16

8.	Yin W, Gorvel L, Zurawski SM, Li D, Ni L, Duluc D, et al. Functional Specialty of CD40 and Dendritic Cell Surface Lectins for Exogenous Antigen Presentation to CD8+ and CD4+ T Cells. EBioMedicine. 2016;5: 46–58. doi:10.1016/j.ebiom.2016.01.029

9.	Rhodes JW, Botting RA, Bertram KM, Vine EE, Rana H, Baharlou H, et al. Human anogenital monocyte-derived dendritic cells and langerin+cDC2 are major HIV target cells. Nat Commun. 2021;12. doi:10.1038/s41467-021-22375-x

10.	Caux C, Massacrier C, Vanbervliet B, Dubois, B, Durand I, Cella M, et al. CD34+ Hematopoietic Progenitors From Human Cord Blood Differentiate Along Two Independent Dendritic Cell Pathways in Response to Granulocyte-Macrophage Colony-Stimulating Factor Plus Tumor Necrosis Factor a: II. Functional Analysis. Blood. 1997;90: 1458–70. Available: http://www.ncbi.nlm.nih.gov/pubmed/9269763

11.	Martínez-Cingolani C, Grandclaudon M, Jeanmougin M, Jouve M, Zollinger R, Soumelis V. Human blood BDCA-1 dendritic cells differentiate into Langerhans-like cells with thymic stromal lymphopoietin and TGF-β. Blood. 2014;124: 2411–20. doi:10.1182/blood-2014-04-568311

12.	Bouschbacher M, Bomsel M, Verronèse E, Gofflo S, Ganor Y, Dezutter-Dambuyant C, et al. Early events in HIV transmission through a human reconstructed vaginal mucosa. AIDS. 2008;22: 1257–66. doi:10.1097/QAD.0b013e3282f736f4

13.	Harman AN, Bye CR, Nasr N, Sandgren KJ, Kim M, Mercier SK, et al. Identification of Lineage Relationships and Novel Markers of Blood and Skin Human Dendritic Cells. J Immunol. 2012;190: 66–79. doi:10.4049/jimmunol.1200779

14.	Botting RA, Bertram KM, Baharlou H, Sandgren KJ, Fletcher J, Rhodes JW, et al. Phenotypic and functional consequences of different isolation protocols on skin mononuclear phagocytes. J Leukoc Biol. 2017;101: 1393–1403. doi:10.1189/jlb.4a1116-496r

15.	Harman AN, Wilkinson J, Bye CR, Bosnjak L, Stern JL, Nicholle M, et al. HIV Induces Maturation of Monocyte-Derived Dendritic Cells and Langerhans Cells. J Immunol. 2006;177: 7103–7113. doi:10.4049/jimmunol.177.10.7103

16.	Harman AN, Bye CR, Nasr N, Sandgren KJ, Kim M, Mercier SK, et al. Identification of Lineage Relationships and Novel Markers of Blood and Skin Human Dendritic Cells. J Immunol. 2013;190: 66–79. doi:10.4049/jimmunol.1200779

17.	Bigley V, McGovern N, Milne P, Dickinson R, Pagan S, Cookson S, et al. Langerin-expressing dendritic cells in human tissues are related to CD1c + dendritic cells and distinct from Langerhans cells and CD141 high XCR1 + dendritic cells . J Leukoc Biol. 2015;97: 627–634. doi:10.1189/jlb.1hi0714-351r

18.	Klechevsky E, Morita R, Liu M, Cao Y, Coquery S, Thompson-Snipes L, et al. Functional specializations of human epidermal Langerhans cells and CD14+ dermal dendritic cells. Immunity. 2008;29: 497–510. doi:10.1016/j.immuni.2008.07.013

19.	Bertram KM, Botting RA, Baharlou H, Rhodes JW, Rana H, Graham JD, et al. Identification of HIV transmitting CD11c+ human epidermal dendritic cells. Nat Commun. 2019;10. doi:10.1038/s41467-019-10697-w

20.	Rhodes JW, Botting RA, Bertram KM, Vine EE, Rana H, Baharlou H, et al. Human anogenital monocyte-derived dendritic cells and langerin+cDC2 are major HIV target cells. Nat Commun. 2021;12. doi:10.1038/s41467-021-22375-x

21.	Crozat K, Guiton R, Guilliams M, Henri S, Baranek T, Schwartz-Cornil I, et al. Comparative genomics as a tool to reveal functional equivalences between human and mouse dendritic cell subsets. Immunol Rev. 2010;234: 177–198. doi:10.1111/j.0105-2896.2009.00868.x

22.	van den Berg LM, Cardinaud S, van der Aar a. MG, Sprokholt JK, de Jong M a. WP, Zijlstra-Willems EM, et al. Langerhans Cell-Dendritic Cell Cross-Talk via Langerin and Hyaluronic Acid Mediates Antigen Transfer and Cross-Presentation of HIV-1. J Immunol. 2015;195: 1763–73. doi:10.4049/jimmunol.1402356

23.	Flamar A-L, Zurawski SM, Scholz F, Gayet I, Ni L, Li X-H, et al. Noncovalent assembly of anti-dendritic cell antibodies and antigens for evoking immune responses in vitro and in vivo. J Immunol. 2012;189: 2645–55. doi:10.4049/jimmunol.1102390

24.	Fenwick C, Croxatto A, Coste AT, Pojer F, André C, Pellaton C, et al. Changes in SARS-CoV-2 Spike versus Nucleoprotein Antibody Responses Impact the Estimates of Infections in Population-Based Seroprevalence Studies. J Virol. 2020;95: 1–12. doi:10.1128/jvi.01828-20

25.	Joo H, Li D, Dullaers M, Kim T-W, Duluc D, Upchurch K, et al. C-Type Lectin-like Receptor LOX-1 Promotes Dendritic Cell-Mediated Class-Switched B Cell Responses. Immunity. 2014;41: 1–13. doi:10.1016/j.immuni.2014.09.009

26.	Planchais C, Hocqueloux L, Ibanez C, Gallien S, Copie C, Surenaud M, et al. Early Antiretroviral Therapy Preserves Functional Follicular Helper T and HIV-Specific B Cells in the Gut Mucosa of HIV-1–Infected Individuals. J Immunol. 2018;200: 3519–3529. doi:10.4049/jimmunol.1701615

27.	Williams WB, Zhang J, Jiang C, Nicely NI, Fera D, Luo K, et al. Initiation of HIV neutralizing B cell lineages with sequential envelope immunizations. Nat Commun. 2017;8: 1–20. doi:10.1038/s41467-017-01336-3

28.	Jardine JG, Kulp DW, Havenar-Daughton C, Sarkar A, Briney B, Sok D, et al. HIV-1 broadly neutralizing antibody precursor B cells revealed by germline-targeting immunogen. Science (80- ). 2016;351: 1458–1463. doi:10.1126/science.aad9195

29.	Dufloo J, Guivel‐Benhassine F, Buchrieser J, Lorin V, Grzelak L, Dupouy E, et al. Anti‐ HIV ‐1 antibodies trigger non‐lytic complement deposition on infected cells . EMBO Rep. 2020;21: 1–16. doi:10.15252/embr.201949351
